# ViceCT and whiceCT for simultaneous high-resolution visualization of craniofacial, brain and ventricular anatomy from micro-computed tomography

**DOI:** 10.1038/s41598-020-75720-3

**Published:** 2020-10-30

**Authors:** Sergi Llambrich, Jens Wouters, Uwe Himmelreich, Mara Dierssen, James Sharpe, Willy Gsell, Neus Martínez-Abadías, Greetje Vande Velde

**Affiliations:** 1 Biomedical Imaging, Department of Imaging and Pathology, Faculty of Medicine, KU Leuven, Herestraat 49 O&N1 box 505, 3000 Leuven, Belgium; 2 Molecular Small Animal Imaging Centre (MoSAIC), KU Leuven, Leuven, Belgium; 3 Centre for Genomic Regulation (CRG, The Barcelona Institute of Science and Technology, 08003 Barcelona, Spain; 4 Universitat Pompeu Fabra (UPF), 08003 Barcelona, Spain; 5 EMBL Barcelona, European Molecular Biology Laboratory, Barcelona, Spain Institució Catalana de Recerca I Estudis Avançats (ICREA), Barcelona, Spain; 6 GREAB-Research Group in Biological Anthropology. Department of Evolutionary Biology, Ecology and Environmental Sciences, BEECA. Universitat de Barcelona, Barcelona, Spain

**Keywords:** Brain, Neurodevelopmental disorders, Bone, Mouse, Disease model

## Abstract

Up to 40% of congenital diseases present disturbances of brain and craniofacial development resulting in simultaneous alterations of both systems. Currently, the best available method to preclinically visualize the brain and the bones simultaneously is to co-register micro-magnetic resonance (µMR) and micro-computed tomography (µCT) scans of the same specimen. However, this requires expertise and access to both imaging techniques, dedicated software and post-processing knowhow. To provide a more affordable, reliable and accessible alternative, recent research has focused on optimizing a contrast-enhanced µCT protocol using iodine as contrast agent that delivers brain and bone images from a single scan. However, the available methods still cannot provide the complete visualization of both the brain and whole craniofacial complex. In this study, we have established an optimized protocol to diffuse the contrast into the brain that allows visualizing the brain parenchyma and the complete craniofacial structure in a single ex vivo µCT scan (whiceCT). In addition, we have developed a new technique that allows visualizing the brain ventricles using a bilateral stereotactic injection of iodine-based contrast (viceCT). Finally, we have tested both techniques in a mouse model of Down syndrome, as it is a neurodevelopmental disorder with craniofacial, brain and ventricle defects. The combined use of viceCT and whiceCT provides a complete visualization of the brain and bones with intact craniofacial structure of an adult mouse ex vivo using a single imaging modality.

## Introduction

Brain dysmorphologies are associated with malformations of the craniofacial skeletal system in 30–40% of congenital disorders ^1–4^. This is the case in holoprosencephaly, micro- and macrocephaly, Apert syndrome, Down syndrome, Williams syndrome, Rett syndrome and Fragile X syndrome ^5–10^, with varying degrees of severity of craniofacial and brain alterations. Experimental mouse models for these disorders are typically used to investigate the mechanisms underlying brain and skull malformations ^1^.

Since the brain and the face have an intimately integrated development and maintain a continuous physical and molecular interplay through common signaling pathways ^2^, it is crucial to analyze the malformations in both systems simultaneously. However, these investigations are hampered by the lack of a technique to visualize the complete anatomy of the brain and the craniofacial bones in a single scan at sufficiently high resolution. Different tomographic methods are available to image the brain or the skull separately.

In vivo methods such as micro-magnetic resonance imaging (µMRI) provide non-invasive tomographic images of the brain anatomy in 3D^11^. However, the resolution (≈ 50 to 100 µm^3^) is sometimes not sufficient to visualize in detail small neuroanatomical structures of interest like the hippocampus ^12^. The resolution is limited by the acquisition times necessary to avoid adverse effects of long exposure to anesthesia ^13^. The alternative is ex vivo imaging, where scanning time is no longer a constraint and microscopic examination provides detailed imaging at high resolution. Nevertheless, a limitation of such ex vivo imaging techniques is the need to dissect the brain from the skull and/or to section the tissue, which disrupts their original 3D structure. Moreover, all ex vivo techniques have the common drawback that the ventricles significantly shrink or collapse after euthanasia ^14–16^, precluding evaluation of ventricular anatomy.

To image the craniofacial complex in 3D, the gold standard is micro-computed tomography (µCT) ^17^. This is a non-destructive technique that produces high-resolution images (≈ 35 to 50 µm isotropic) of mineralized tissue using short scanning times and low radiation exposure in vivo ^18^, as well as ultra-high resolution images (≈ 1 to 9 µm) using longer exposure ex vivo ^19^.

To visualize the brain and the skull simultaneously, the most common approach is to scan each structure separately using µMRI for the brain and µCT for the skull, and then co-registering both scans ^20,21^. However, this results in a time and cost-demanding pipeline requiring multiple acquisitions, expertise in and access not only to both imaging techniques, but also to advanced image processing and co-registration software. In this dual-modality setup, the need of µMRI to visualize the brain is associated with manifold higher infrastructure and operational costs than µCT.

Diffusible iodine-based contrast-enhanced computed tomography (diceCT) could provide an alternative to image the brain and the craniofacial complex within a single acquisition. DiceCT is a time and cost efficient ex vivo imaging procedure that uses iodine as contrast agent, thereby allowing the visualization of both skeletal and soft tissues at the high resolution from a µCT scan ^22–27^. However, diceCT procedures for adult mice only manage to deliver the contrast to the brain at the expense of the integrity of the craniofacial complex. In the approaches where diceCT has been applied to visualize the mouse and rat brain, the skull was completely removed ^28–31^. In others, where adult mouse brains were imaged within the skull, several parts of the craniofacial complex were removed to enhance the diffusion of iodine-potassium iodide (I_2_KI) into the brain ^32^. Alternative protocols have explored the use of selectively perfusable iodine-based contrast-enhanced computed tomography (spiceCT) to administer contrast to the entire mouse body leaving the entire anatomy intact including the craniofacial structure, but this strategy does not manage to provide contrast into the brain ^33^. Imaging protocols using other contrast agents such as phosphotungstic acid (PTA) have enabled the visualization of bone and soft tissue from µCT scans with minimal shrinkage ^34^, but have not yet been optimized for adult mice, are more costly and require more elaborated sample processing. Finally, the brain ventricles are not visible in any of these approaches, which implies an extra limitation for the neurodegenerative and neurodevelopmental disorders research fields, where the ventricular anatomy or volume are important biomarkers ^35–38^.

We therefore set out to develop a novel approach that allows to simultaneously visualize and phenotype the anatomy of the brain or the ventricles and craniofacial structures, from a single modality using high-resolution µCT acquisition. We have tested different protocols for contrast delivery, such as transcardial perfusion and diffusion of iodine solution to obtain homogeneous contrast into the brain parenchyma while maintaining the craniofacial structures intact. In addition, we have developed a new technique that uniquely allows visualization of the mouse brain ventricles without collapsing, maintaining intact the brain, head and even whole body. Finally, to demonstrate the potential of these novel imaging techniques we applied them in the Ts65Dn mouse model of Down syndrome, an example of a congenital disorder where the craniofacial complex, the brain, and the ventricles are simultaneously affected ^10,39–44^.

## Results

### Transcardial perfusion for iodine-based contrast delivery to the brain

First, we explored different approaches to deliver the contrast to the brain via transcardial perfusion while modulating blood–brain barrier (BBB) permeability in wildtype (WT) mice. In all our experiments, Lugol’s solution was used as the source of iodine. We assessed different protocols adjusting the order, timing and proportion of perfused reagents (Supplementary Table S1). We then scanned the adult mice with whole-body µCT.

**Figure 1:**
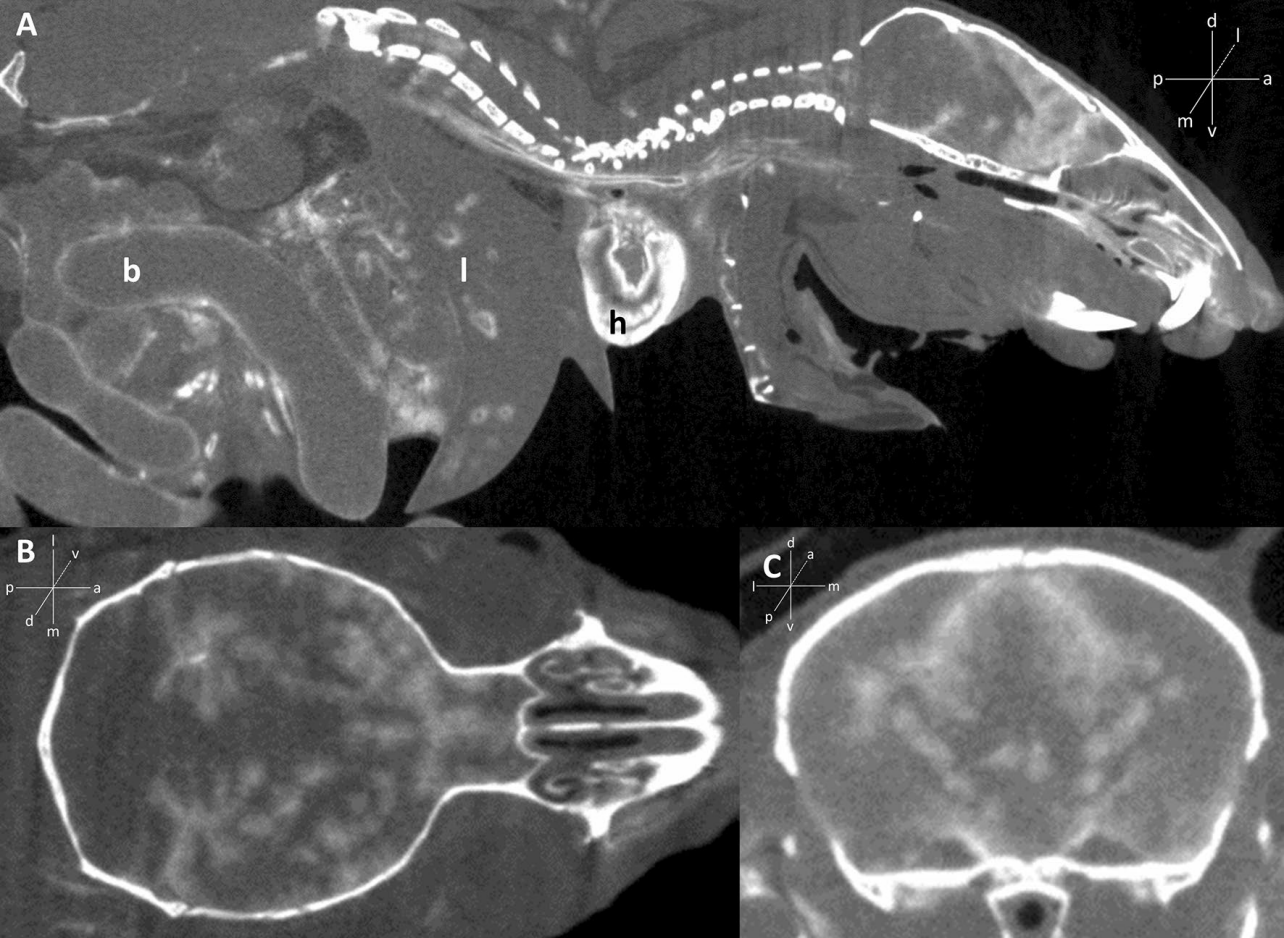
Whole-body µCT upon transcardial perfusion of Lugol’s solution after blood–brain barrier disruption results in contrast enhancement in body organs and non-homogeneous contrast in the brain. **(A)** Sagittal view of the upper mouse body, perfused according to Supplementary Table S1 protocol H. Contrast enhancement could be detected in the brain and several body organs, such as bowels (b), liver (l) and heart (h). **(B)** Axial and **(C)** coronal µCT view of the head, visualizing the skull and patchy contrast enhancement in the brain parenchyma. The scans reveal that the skull is visible and the contrast was delivered into the brain, but the staining was not homogeneous.

The whole craniofacial complex was visible in all the µCT scans, but none of the transcardial perfusion protocols resulted in homogeneous brain contrast (Supplementary Figure S1). Perfusing the Lugol’s solution for 50 min and fixing the tissue by incubating the sample in 4% paraformaldehyde (PFA) for 48 h did not provide contrast in the brain, but increased contrast in soft tissues such as cardiac muscles, pericardial structures and lungs (Supplementary Table S1 G; Supplementary Figure S2). We could achieve patchy brain contrast when perfusing first with Lugol’s solution during 20 min (Supplementary Table S1 F; Supplementary Figure S1 F) and when disrupting the BBB with mannitol (Fig. 1; Supplementary Table S1 H).

### Whole head iodine-based contrast-enhanced computed tomography (whiceCT)

In a second approach, we developed an iodine-diffusion technique to visualize the brain parenchyma with minimal shrinkage and without removing the facial skeleton. We explored the optimization of different parameters to facilitate the contrast diffusion into the mouse brain. We removed the external soft tissue of the severed mouse heads and drilled two burr holes on the parietals to facilitate diffusion. Then, the heads were incubated in Lugol’s solution for different periods of time and at different refreshment rates. To establish the optimal staining conditions, we scanned the samples several times during this process with µCT.

**Figure 2:**
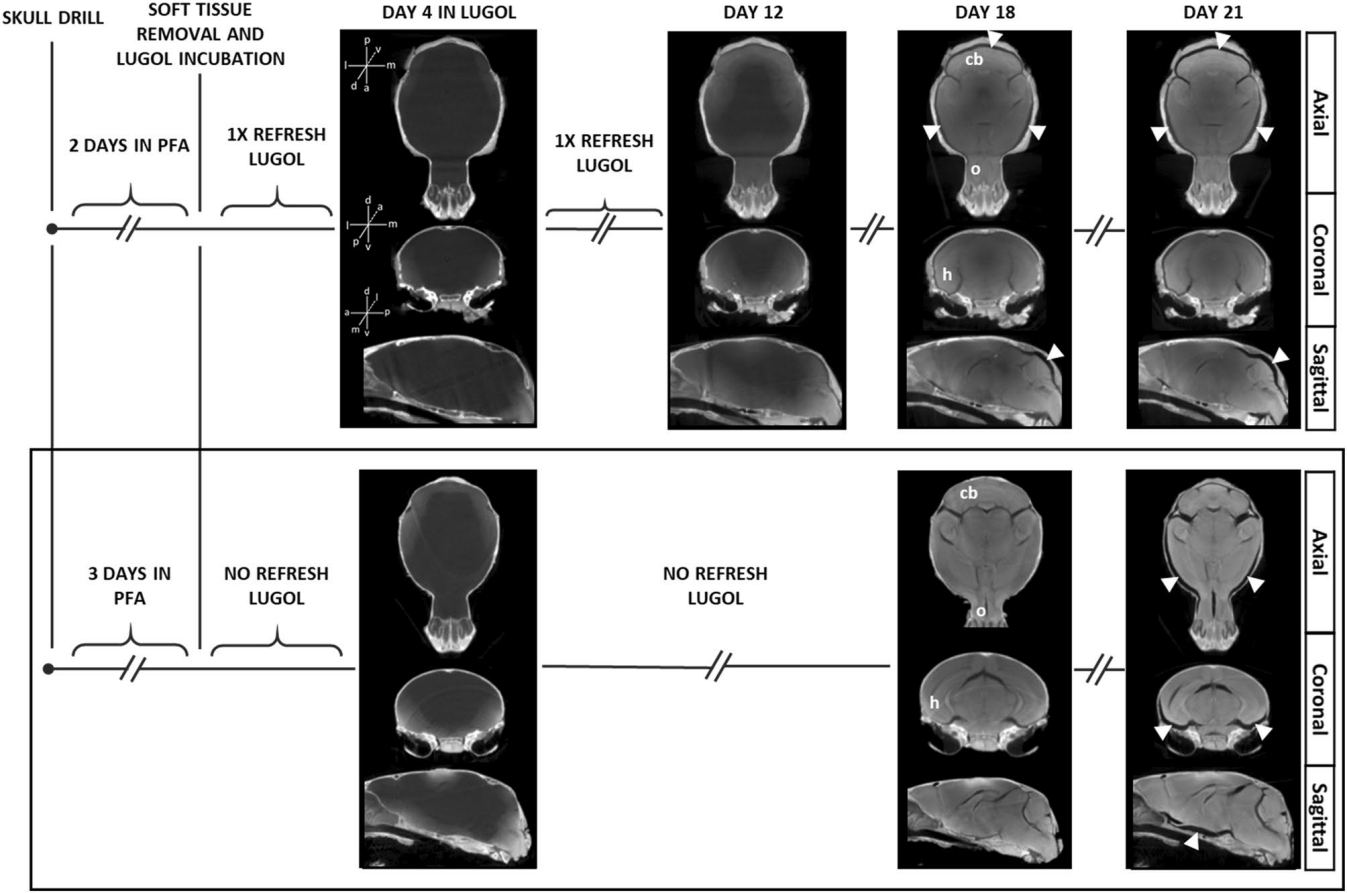
WhiceCT results across the sample processing pipeline. Top row: µCT Scans obtained after the mouse heads were immersed in Lugol’s solution for 4, 12, 18 and 21 days; with the Lugol’s solution refreshed as indicated in the timeline. µCT at day 4 confirmed correct positioning of drill holes and contrast diffusing into the brain parenchyma. By day 18, contrast enhancement was sufficient to visualize brain structures such as the cerebellum (cb), olfactory bulbs (o) and hippocampus (h), but some shrinkage was observed (white arrowheads). Incubating the sample until day 21 did not further increase the contrast and worsened the shrinkage. In a subsample of mouse heads, the Lugol’s solution was not refreshed at day 2, which did not result in any observable difference (not shown). Bottom row: Scans obtained after the mouse heads were immersed in Lugol’s solution for 4, 18 and 21 days with the Lugol’s solution only renewed at each scan time. µCT showed incomplete contrast diffusion into the brain by day 4. By day 18, minimal shrinkage was observable around the cerebellum while the contrast was optimal to visualize the cerebellum, the olfactory bulbs and the hippocampus among other brain structures. Finally, incubating the samples for three more days did not further improve contrast and caused further shrinkage. The bottom framed protocol is the optimal proposed pipeline for visualizing the intact craniofacial structures and brain parenchyma with minimal shrinkage and optimal contrast from a single µCT at day 18.

In a first optimization round, the mouse heads were fixed in PFA during two days and incubated with 30 mL of Lugol’s solution that was renewed six times during 21 days (at days 2, 4, 9, 12, 18 and 21) (Fig. 2 top row). The whiceCT scans produced detailed images of both the whole skull and the brain parenchyma after 18 days of sample incubation. The contrast enhancement was sufficient to visualize brain structures such as the cerebellum, olfactory bulbs and hippocampus. However, the brain presented with significant shrinkage (Fig. 2 top row, white arrows). Incubating the samples until day 21 improved the contrast at the core of the brain, but worsened shrinkage (Fig. 2 top row).

To minimize brain shrinkage, we increased the fixation time in PFA from two to three days, incubated the samples within a higher volume of Lugol’s solution (from 30 to 45 mL) and limited changing the Lugol’s solution only to the scanning days (days 4, 18 and 21) (Fig. 2 bottom row). The µCT scans showed that by day 18 the contrast in the different brain structures (cerebellum, olfactory bulbs and hippocampus) optimally improved while the brain shrinkage was minimal (Fig. 2 bottom row; Video 1 with 3D visualization of a whiceCT scan). Incubating the samples until day 21 improved the contrast at the most inner regions of the brain, but worsened shrinkage (Fig. 2 bottom row, white arrows), indicating that incubating the samples until day 18 is the most optimal for improved contrast and reduced shrinkage.

We have named this new optimized technique as “whole head iodine-based contrast-enhanced CT” or “whiceCT”. WhiceCT is a useful method to visualize the brain parenchyma with minimal shrinkage together with the craniofacial structures in a single µCT scan.

**Figure 3:**
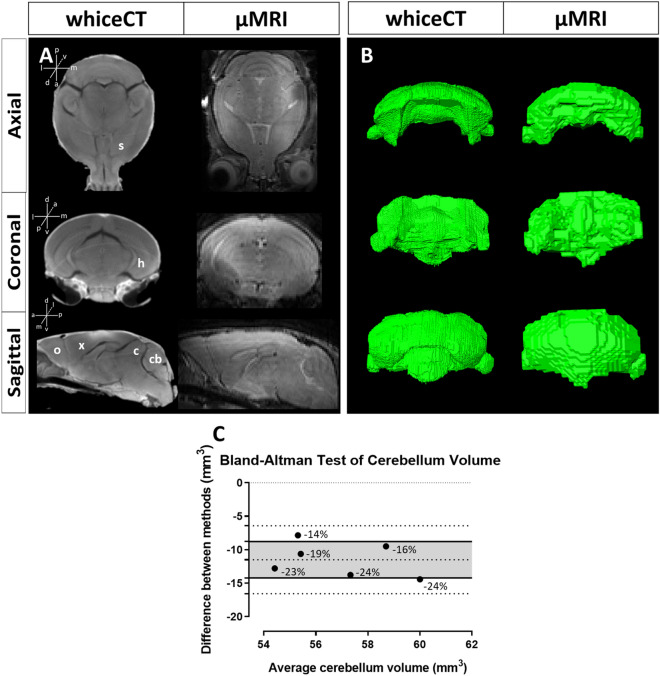
WhiceCT compared to in vivo µMRI. **(A)** Reconstructed images of whiceCT (left) and in vivo µMRI (right). Similar edge definition can be observed for both µCT as µMRI in the brain parenchyma in the axial (top), coronal (middle) and sagittal (bottom) planes and the same structures can be visualized, except for the ventricles, as they are collapsed in the ex vivo scan. For example, the striatum (s), hippocampus (h), isocortex (x), thalamus (t), olfactory bulbs (o), cerebellum (cb) and colliculi (c) are visible. **(B)** Volume renderings of the cerebellum segmented from whiceCT (left) and in vivo µMRI (right). Note the similarity in shape and the difference in voxel size. **(C)** Bland–Altman test of the agreement between cerebellum volume obtained with whiceCT and µMRI (n = 6). The densely dotted line at 0 in the Y axis represents the line of equality. Upper and lower loosely dotted lines represent the 95% limits of agreement. The central loosely dotted line represents the bias. Shaded area represents 95% confidence interval limits for the bias. The average shift represented − 20.21% of the mean cerebellum volume, calculated as the ratio between the bias (11.49 mm^3^) and the mean volume of the cerebellum (56.86 mm^3^). The differences between the two measurements over the mean cerebellum volume, calculated as the ratio between the 95% limits of agreement of the bias (− 6.42 mm^3^ and − 16.56 mm^3^) and the mean volume of the cerebellum (56.86 mm^3^), can range from − 11.29% to − 29.12%. Negative values indicate lower volumes obtained with µCT. The percentage of difference between methods is shown for each comparison.

To cross-validate the whiceCT protocol with the gold standard for brain imaging, we scanned six WT mice with in vivo µMRI and subsequently performed whiceCT. Upon visual comparison, the tomographic images showed similar contrast and edge definition of brain anatomical regions in both imaging techniques (Fig. 3A). Except for the ventricles, which were not visible from the whiceCT scan due to their collapse after sacrifice, the same brain structures could be identified in whiceCT and µMR scans without obvious signs of shrinkage, such as the isocortex, hippocampus, striatum, colliculi, olfactory bulbs and cerebellum (Fig. 3A, Supplementary Figure S3). Additionally, whiceCT scans allowed to visualize the skull, which was not visible from the µMRI. WhiceCT presented minimal shrinkage at the anterior ventral part of the brain, visualized as a minimal gap between the brain and the skull (Supplementary Figure S3). More extended shrinkage was only detected in some mice at the level of the cerebellum. A possible explanation could be that this posterior region of the brain is closest to the neck surgical cut used to severe the heads and was therefore more exposed to Lugol’s than the internal and anterior regions of the brain (Supplementary Figure S3).

To perform more specific comparisons regarding the brain region where we observed shrinkage, we segmented the cerebellum from both the µMRI and µCT scans and compared the resulting 3D reconstructions regarding their shape, resolution and volume. The segmented cerebellums were similar in shape (Fig. 3B). The reconstruction from the whiceCT protocol showed increased detail due to the higher resolution of µCT (51.62 µm for 11 min scan time) as compared to the resolution of the in vivo µMRI (156 µm for 20 min scan time) (Fig. 3B). To further evaluate the agreement between whiceCT and µMRI, we performed a Bland Altman test that assessed the differences in cerebellar volume measured by the two methods. The results revealed that the differences were consistent for low and high cerebellar volumes, as the points were equally distributed along the x-axis (Fig. 3C). However, the Bland Altman test indicated that the cerebellum volume estimated from whiceCT scans was consistently lower than the volume measured from µMRI (Fig. 3C). On average, there was a difference between methods of − 20.21% of the mean cerebellar volume. Individual differences between methods could range from − 11.29 to − 29.12% of the mean cerebellar volume, considering the 95% confidence intervals. We found a maximum volume difference of − 14.41 mm^3^, which represented 24.34% of the mean cerebellum volume (Fig. 3C). These differences in cerebellar volume could possibly be attributed to a certain degree of tissue shrinkage during the sample processing procedure. However, some differences could also arise from differences in the resolution in the two scanning methods, as µCT has higher resolution than µMRI, produces smaller voxel sizes and this may also affect the segmentation. Overall, the results from the Bland Altman test performed in the brain region most affected by shrinkage provide a reliable quantification of the maximum difference in volume that could be expected between µMRI and whiceCT.

**Figure 4:**
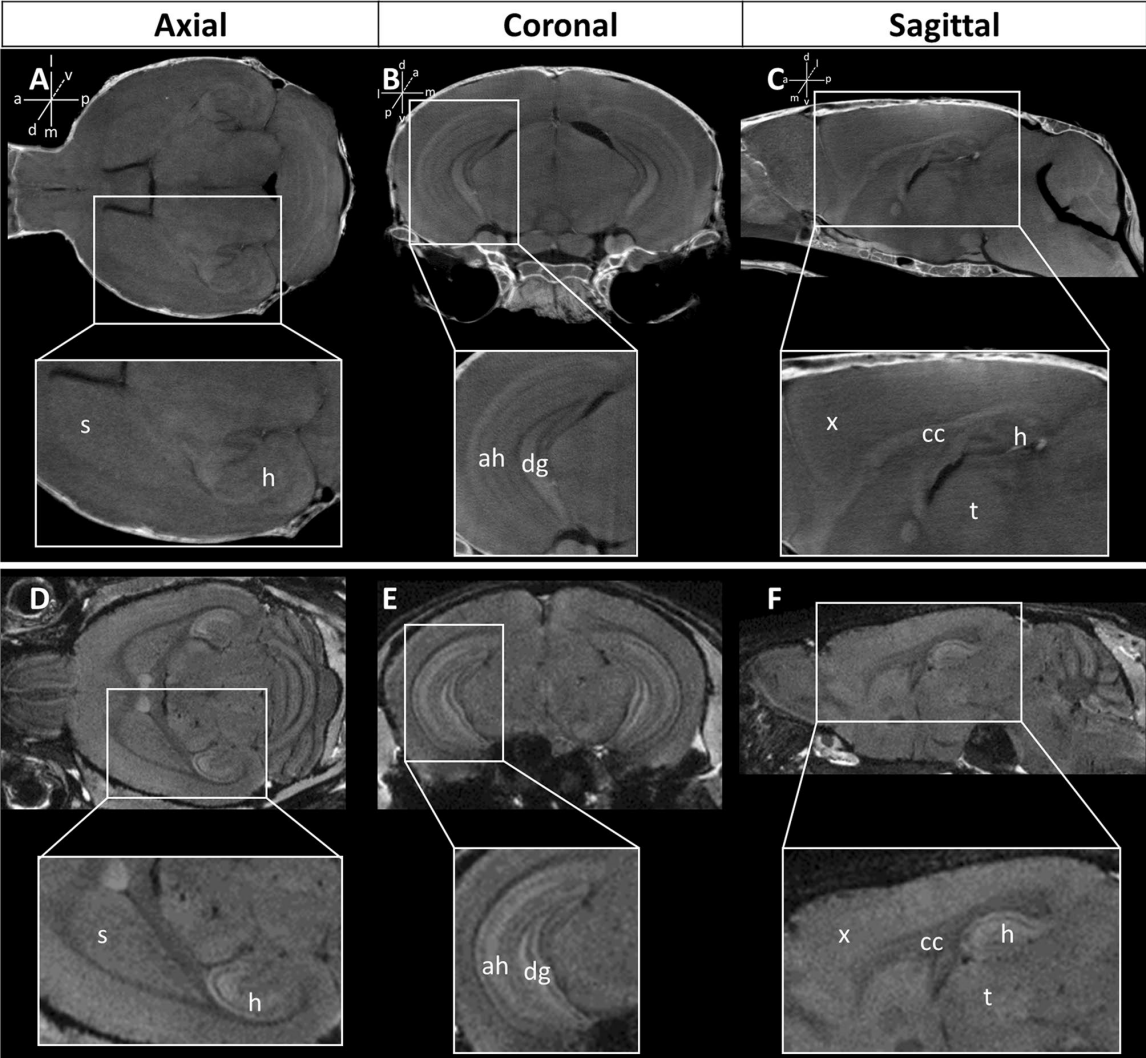
Images obtained with ultra-high resolution whiceCT (9 µm) compared with ex vivo µMRI (62.5 µm). Top: images obtained with an ultra-high-resolution whiceCT, with zoom-in to detailed structures of interest, such as the striatum (s), hippocampus (h), Ammon’s horn (ah), dentate gyrus (dg), isocortex (x), corpus callosum (cc) and thalamus (t). Bottom: images obtained using a contrast-enhanced µMRI with similar zoom-in details. Panels A and D: axial planes, panels B and E: coronal planes and panels C and F: sagittal planes.

Finally, our whiceCT protocol can provide ultra-high-resolution images of the brain and skull in comparison with a high-resolution ex vivo µMRI scan that only visualizes the brain. To illustrate this potential, a mouse head was scanned with whiceCT at an isotropic resolution of 9 µm in 3 h and 3 min and was compared with another mouse head scanned with gadolinium-enhanced ex vivo µMRI at an isotropic resolution of 62.5 µm in 12 h and 17 min. Qualitative comparison of the tomographic images of the ultra-high resolution whiceCT (Fig. 4 top) and contrast-enhanced ex vivo µMRI (Fig. 4 bottom), showed that both ultra-high resolution whiceCT and ex vivo µMRI showed the hippocampus and the striatum in the axial view (Fig. 4A,D). In the coronal view (Fig. 4B,E), both techniques were able to visualize the hippocampus and distinguish the Ammon’s horn and dentate gyrus. In the sagittal view (Fig. 4C,F) both images show the isocortex and corpus callosum with the MR images showing better contrast in the hippocampus but the µCT images having better contrast in the thalamus.

Overall, the whiceCT protocol produced detailed images of the entire craniofacial structure as well as brain structures at shorter scanning times as compared with in vivo and ex vivo µMRI. Moreover, smaller structures could be visualized increasing the resolution and acquiring more projections resulting in a scan time of 3 h and 3 min, achieving higher resolution and less noise than ex vivo µMRI scan in 12 h and 17 min (Fig. 4).

### Ventricle injection contrast-enhanced CT (viceCT)

As whiceCT and ex vivo µMRI are techniques where imaging is performed after the mice have been euthanized, the ventricles collapse due to the loss of cerebrospinal fluid pressure and, thus, the original morphology of the brain ventricles is lost. To enable the evaluation of ventricular morphology, we developed a novel protocol to visualize the ventricles before they collapse by directly injecting the contrast into the ventricles under terminal anesthesia. We explored an optimal approach to perform stereotactic injections of the contrast into the lateral brain ventricles of terminally anesthetized mice by changing the volume of Lugol’s solution and the injection location. To assess whether these staining protocols resulted in homogeneous contrast in the ventricular system immediately after contrast injection, the mice were scanned with µCT with optimized scanning parameters (Supplementary Table S2).

**Figure 5:**
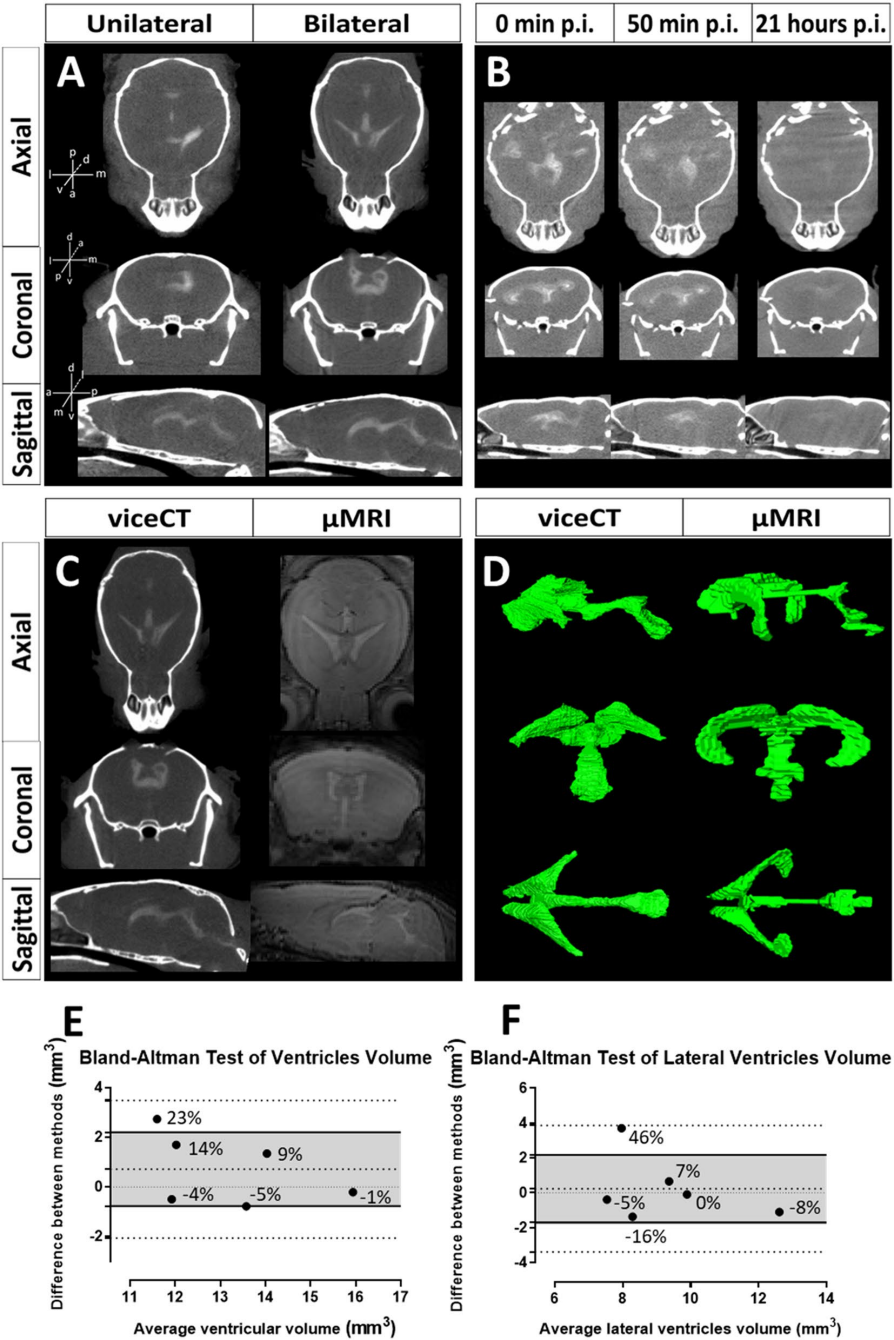
Qualitative and statistical evaluation of the ventricular system obtained with viceCT compared to in vivo µMRI. **(A)** Images obtained using a unilateral (left column) and a bilateral (right column) ventricular injection of Lugol’s solution, where enhanced contrast can be observed in the ventricles in the axial (top row), coronal (middle row) and sagittal (bottom row) planes. **(B)** Time course visualization of a mouse brain processed with viceCT at 0 min, 50 min and 21 h post injection (p.i.). In the scan acquired 0 min p.i., the ventricles were visible and clearly distinguishable from the brain parenchyma. In the scan acquired 50 min p.i, the delineation of the ventricles was less pronounced. In the scan acquired 21 h p.i., the contrast was no longer detectable on µCT, indicating that the ventricular contrast enhancement faded over time. **(C)** Images obtained using viceCT (left column) and in vivo µMRI (right column) showed similar contrast enhancement in the ventricles, observed in the axial (top row), coronal (middle row) and sagittal (bottom row) planes. **(D)** 3D surface rendering of the segmented ventricles obtained from viceCT (left column) and in vivo µMRI (right column) showed similar shapes, but detailed inspection revealed differences in the shape of the ventral part of the lateral ventricles, the fourth ventricle and the cerebral aqueduct. **(E)** Bland–Altman test representing the difference in ventricle volume obtained with viceCT and µMRI (n = 6). Densely dotted line at 0 in the Y axis represents the line of equality. Upper and lower loosely dotted lines represent the 95% limits of agreement. Central loosely dotted line represents the bias. Shaded area represents 95% confidence interval limits for the bias. The relative average bias represented 5.39% of the mean ventricle volume, calculated as the ratio between the average bias (0.71 mm^3^) and the mean volume of the ventricle (13.18 mm^3^). The differences between the two measurements over the mean ventricle volume, calculated as the ratio between the 95% limits of agreement of the bias (− 2.06 mm^3^ and 3.48 mm^3^) and the mean volume of the ventricle (13.18 mm^3^), can range from − 15.62% to 26.40%. Negative values indicate lower volumes obtained with µCT. The percentage of difference is shown for each comparison. **(F)** Bland–Altman test representing the difference in the lateral ventricles volume obtained with viceCT and µMRI (n = 6). The relative average bias represented 2.59% of the mean ventricle volume, calculated as the ratio between the average bias (0.24 mm^3^) and the mean volume of the lateral ventricles (9.28 mm^3^). The differences between the two measurements over the mean lateral ventricles volume, calculated as the ratio between the 95% limits of agreement of the bias (− 3.38 mm^3^ and 3.86 mm^3^) and the mean volume of the lateral ventricles (9.28 mm^3^), can range from − 36.42% to 41.59%. Negative values indicate higher volumes obtained with µMRI. The percentage of difference between methods is shown for each comparison.

First, we performed a unilateral stereotactic injection of 100 µL of Lugol’s solution into the right lateral ventricle of the mice. Although the brain ventricles were visible on the µCT scans, the contrast was not confined to the ventricular system and the high pressure during contrast administration could have altered the ventricular anatomy (Supplementary Figure S4). With an injected volume of Lugol’s solution of 50 µL, the contrast remained confined within the right lateral ventricle, the third and fourth brain ventricles and the cerebral aqueduct and no anatomical alterations could be observed (Fig. 5A, left). However, the contrast was not homogeneously distributed in the left lateral brain ventricle. To visualize simultaneously both the right and left lateral brain ventricles, we next performed a bilateral stereotactic injection of 40 µL of Lugol’s solution into each of the left and right lateral ventricles. The resulting µCT showed that the contrast was confined and uniformly distributed in both lateral ventricles, the third and the fourth ventricles and the cerebral aqueduct (Fig. 5A, right; Video 2 with 3D visualization of a viceCT scan). Moreover, the whole-body scan of the intact mouse allowed to evaluate the ventricle morphology in context of the cranium and the whole skeleton. We named this optimized technique as ‘ventricle injection contrast-enhanced CT’ or ‘viceCT’.

We quantified the contrast enhancement produced by iodine in the ventricles by viceCT as the difference in radiodensity of the brain parenchyma and the ventricles. The average radiodensity in the brain parenchyma was 180 ± 23 HU and 1,311 ± 304 HU in the brain ventricles, which resulted in a contrast enhancement of 1,131 ± 281 HU (n = 6) (Supplementary Table S4).

To evaluate the stability of the contrast over time for viceCT, we performed a bilateral ventricular stereotactic contrast injection and scanned the mouse at three different time points: immediately after injection, 50 min after injection, and 21 h after injection. Qualitative evaluation of the scans showed that the contrast faded over time and was no longer detectable after 21 h (Fig. 5B). The quantitative evaluation confirmed that the ventricles’ radiodensity decreased from 1,573 HU in the first timepoint to 1,487 HU in the second and to 861 HU in the third (Supplementary Table S4). This indicates that the mice should be scanned within one hour after stereotactic injection to benefit from maximal contrast enhancement.

To compare the image quality and the level of contrast enhancement of viceCT and µMRI, we computed the signal-to-noise ratio (SNR) and the contrast-to-noise ratio (CNR) of mouse heads imaged with µMRI and subsequently with viceCT (Fig. 5C; Supplementary Table S4). In the brain parenchyma, the SNR was comparable for viceCT (24.87 ± 6.74, n = 6) and µMRI (28.66 ± 10.02), whereas in the ventricles the mean SNR was lower for viceCT (21.30 ± 5.04) than for µMRI (41.16 ± 12.69). Nevertheless, all SNR values were high above 5 and thereby met the Rose criterion, which is currently the most used reference detectability measure^45,46^. Moreover, the CNR for the ventricles was superior for viceCT (22.47 ± 2.95) as compared to µMRI (12.50 ± 4.24), and overall high for both modalities, making it possible to reconstruct the morphology of the brain ventricles from both types of scans.

We next cross-validated the ability to segment the ventricles from viceCT using in vivo µMRI as a reference. Gross visual comparison of the brain ventricles showed overall similar shapes, but more detailed inspection revealed differences in the shape of the brain ventricles segmented from viceCT and in vivo µMR scans (Fig. 5D). Whereas µMRI showed a more complete segmentation of the ventral part of the lateral ventricles, viceCT provided greater detail in the reconstructions of the fourth ventricle and cerebral aqueduct. Moreover, due to smaller voxel size, viceCT provided better spatial resolution, resulting in more detailed images within shorter scan time (Fig. 5D).

Finally, we investigated the agreement between the volumes of the entire ventricular system and the lateral ventricles segmented with µMRI and viceCT using a Bland Altman test. The results indicated that the ventricular and lateral ventricles’ volume estimated from viceCT and µMRI scans were similar and consistent for low and high ventricular volumes (Fig. 5E,F). On average, there was a non-significant difference between methods of 5.39% of the mean ventricle volume, as the line of equality fell within the 95% confidence interval of the bias. Individual differences between methods could range from − 15.69 to 26.40% of the mean ventricle volume, considering the 95% confidence intervals. We found a maximum volume difference of 2.73 mm^3^, which represented 20.71% of the mean ventricle volume (Fig. 5E). For the lateral ventricles, the average difference of the mean lateral ventricles’ volume of 2.59% was not significant. Individual differences between methods could range from 36.42 to 41.59% of the mean lateral ventricles’ volume and the maximum volume difference found was 3.71 mm^3^ (40% of the mean lateral ventricles’ volume) (Fig. 5F). This higher variation is mainly due to the discrepancy in measurements between viceCT and µMRI in only one mouse, as the other five mice presented low differences between viceCT and µMRI in the estimation of the lateral ventricles’ volume and thus consistent results between viceCT and µMRI (Fig. 5F). Our results thus suggest that viceCT is a useful technique for the analysis of both the entire ventricular system and the lateral ventricles.

### Comparison of viceCT and whiceCT for skull analysis

**Figure 6:**
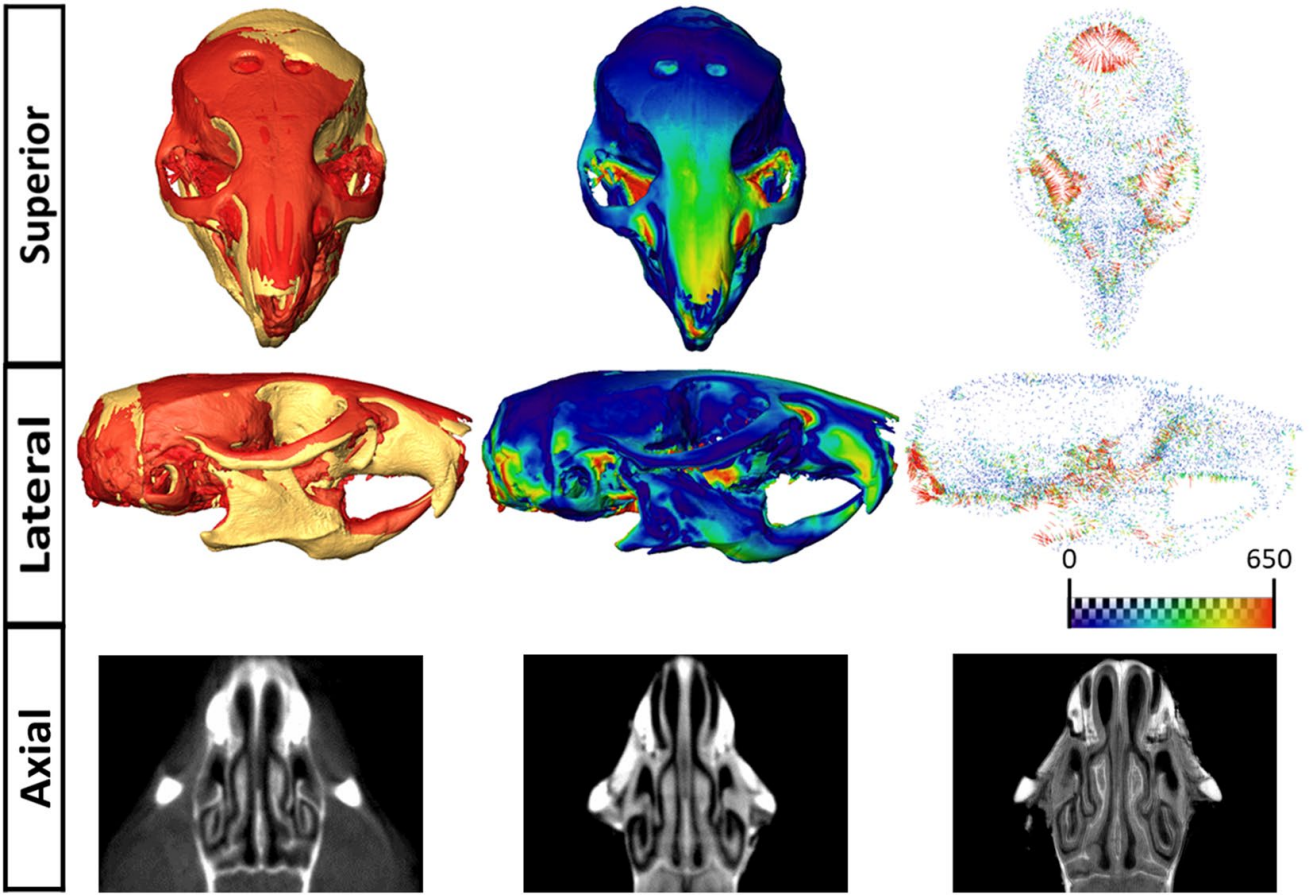
Comparison of the skull obtained from whiceCT and viceCT. Top and middle: comparison of the 3D skull reconstructions segmented from viceCT and whiceCT scans of the same mouse. Left: superposition by rigid registration of the two reconstructions, as obtained with viceCT (yellow) and whiceCT (red). Central: heatmap showing the differences between a viceCT and a whiceCT scans, with red areas indicating where the largest differences occur. Right: differences between viceCT and whiceCT displayed as a vector map. Bottom: axial tomographic images of the snout of a mouse obtained with viceCT (left) whiceCT (middle) and ultra-high resolution whiceCT (right).

We have developed two novel techniques that allow visualizing at high resolution the brain ventricles (viceCT) and the brain parenchyma (whiceCT) together with the skull from a µCT scan. Both techniques allow to visualize the morphology of the skull. The comparison between the 3D surface skull reconstructions obtained with viceCT and whiceCT showed that for most regions of the skull, viceCT and whiceCT scans produced similar outcomes, with minimal differences between them (Fig. 6, top and middle). More substantial differences were only localized in those regions where some soft tissue was still attached to the skull in the whiceCT scan (Fig. 6, top and middle). The comparison of the tomographic images of the snout showed that a thin, low-density bone such as the ethmoid cannot be clearly visualized using whiceCT, but is visible in the viceCT scan and clearly defined in the ultra-high resolution whiceCT image (Fig. 6, bottom). These results demonstrate that both techniques can be used for the evaluation of the skull morphology and underscore that for detailed qualitative and quantitative skull analysis such as with Geometric Morphometrics, the use of whiceCT requires careful manipulation of the sample to completely extract the soft tissue without damaging or scratching the skull. Therefore, the use of viceCT is recommended to perform skull morphometric analyses, as a more practical, precise and reliable procedure to image the skull, as we illustrate in the next section.

### Applications of viceCT and whiceCT

To show the capability of our approach towards phenotyping mouse models of conditions that require integrated evaluation of the craniofacial and brain morphology including the ventricles, we applied the combination of viceCT with whiceCT to evaluate ventricular anatomy and whole-brain anatomy within an intact craniofacial skeleton in a trisomic (TS) mouse model of Down syndrome ^47^.

Specifically, we applied both viceCT and whiceCT to the same mice, to assess whether these combined techniques are able to pick up major phenotypic differences induced by genetic differences in euploid and trisomic mice ^10,39–44^, as well as differences induced by pharmacological modulation by green tea extracts containing epigallocatechin-3-galate (GTE-EGCG). GTE-EGCG has been used as a modifying agent of the morphology of the brain, ventricles and skull, as it is known to mediate brain and craniofacial development in this mouse model for Down syndrome ^41,48–50^. An image for each condition is shown in Supplementary Figure S5.

**Figure 7:**
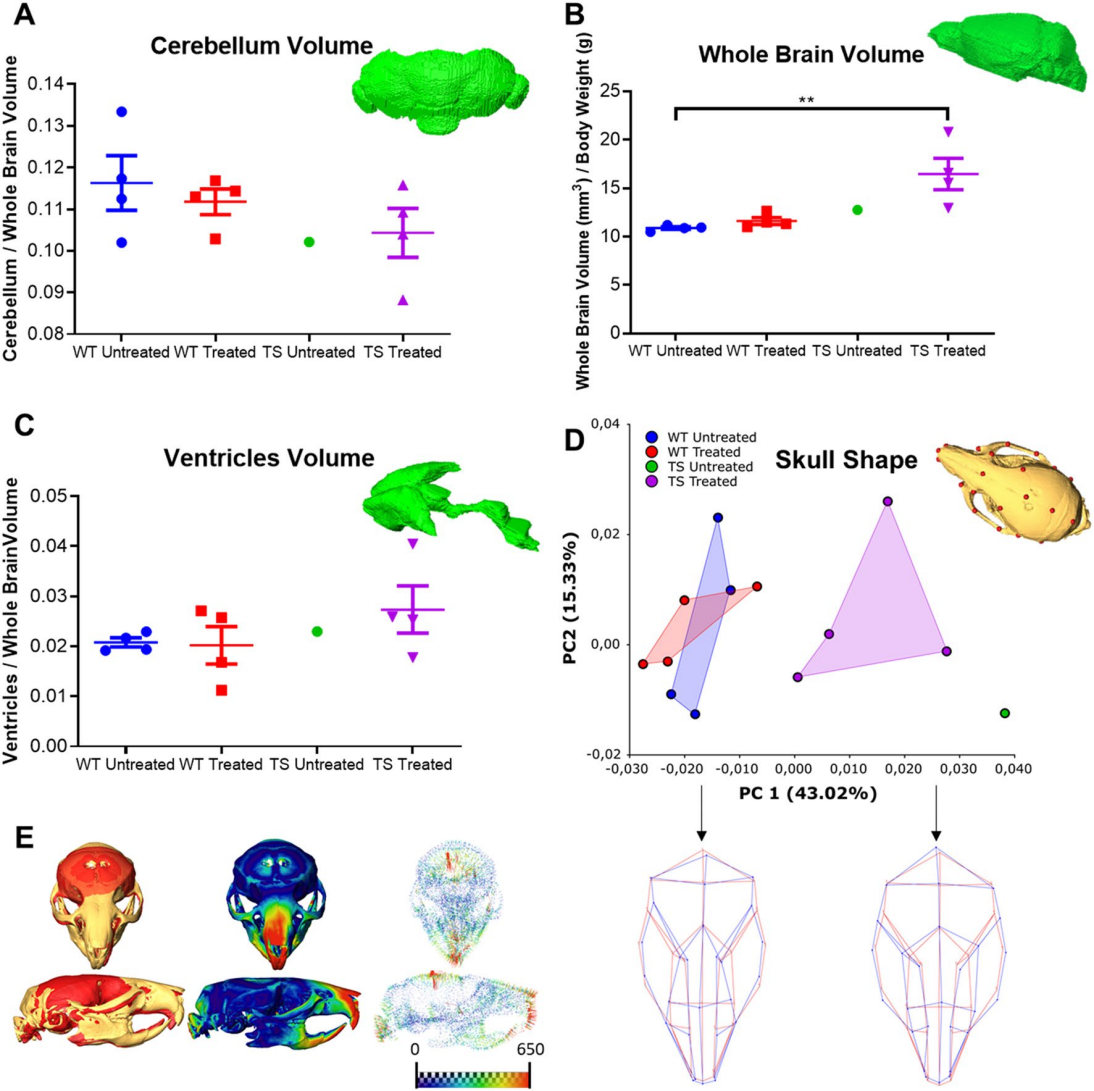
ViceCT combined with whiceCT enable quantification of brain regions’ volume and skull shape to evaluate genotype and pharmacological effects in a Down syndrome mouse model. **(A)** WhiceCT-based 3D surface rendering of the cerebellum and grouped scatter plot comparing the cerebellar volume normalized to the whole brain volume of wildtype (WT) and trisomic (TS) mice, pharmacologically modulated or not with GTE-EGCG. **(B)** ViceCT-based 3D surface rendering of the brain and grouped scatter plot of the relative whole brain volume. **(C)** ViceCT-based 3D surface rendering of the ventricular system and grouped scatter plot of the relative ventricle volume. Error bars represent standard error of the mean in all scatter plots. **(D)** Scatter plot assessing skull shape variation from a Principal Component Analysis (PCA) based on the landmarks described in Supplementary Table S3. In the scatterplot of the first two PC axes, which together explain 58.35% of the variation, each point represents the skull shape of one individual and experimental groups of mice are outlined using convex hulls, except for the TS untreated mouse. Close proximity between groups indicates similar skull phenotypes, while separation indicates dissimilarity. Wireframes displaying skull morphologies along PC1 of the PCA analysis based on the global skull configuration of landmarks (Supplementary Figure S6). Dark blue wireframes represent the morphology associated with the specimens located on the negative (WT mice, left) or positive (TS mouse, right) extreme of PC1 in comparison to the mean shape of the sample (red wireframe). Note that the wireframes are exaggerated and correspond to the shapes that would be seen at values of 0.1 and − 0.1 in PC1 to better illustrate the differences in shape. **(E)** Comparison of the 3D surface rendering of a WT and a TS mouse segmented from viceCT scans. Left images show two representative skulls overlaid by rigid registration with the WT mouse in yellow and the TS mouse in red, highlighting that TS mice (red) present with shorter and wider skulls (brachycephaly) and less developed faces (facial retraction), which correspond to the clinical features associated with Down syndrome in humans. Central images display a heatmap showing the dissimilarities between a WT and a TS mouse with red areas representing higher differences. Right images display a vector map showing in red the largest dissimilarities between WT and TS mice. WT Untreated n = 4, WT Treated n = 4, TS Untreated n = 1, TS Treated n = 4.

WhiceCT scans visualized single brain structures, such as the hippocampus, the cerebellum, the isocortex and the olfactory bulbs. The efficient contrast enhancement allowed us to segment the cerebellum for comparison among WT and TS mice, as it is a structure known to be severely affected in trisomic Down syndrome mouse models ^51–53^, and to assess whether GTE-EGCG produced any further difference in these groups of mice. Both untreated and treated TS mice showed relatively smaller cerebellar volume as compared with WT euploid mice, although the differences did not reach the significance level considering the high variation in cerebellar volume in all the mouse groups and the small sample sizes (Fig. 7A).

ViceCT scans visualized the outline of the ventricles and the brain, which was clearly defined by the endocranial space of the skull, allowing straightforward segmentation of these structures. ViceCT detected a significant increase in the whole brain volume of the TS treated group as compared with the WT untreated group, showing the capability of this method to detect differences due to genotype and pharmacological intervention (Fig. 7B). Moreover, viceCT detected that the WT and TS treated groups showed higher variation in the ventricular volume than the untreated WT mice (Fig. 7C).

To perform a quantitative skull shape comparison between experimental mouse groups, we used the high resolution viceCT scans, for the reasons explained above. We recorded the 3D coordinates of 27 anatomical landmarks on the 3D skull reconstructions resulting from the viceCT scans. Following a Geometric Morphometric approach, we explored skull morphological variation using a Principal Component Analysis (PCA) after the Procrustes superimposition of the landmark coordinates. Results showed that WT and TS mice were separated in the morphospace along PC1, indicating that most skull morphological variation in the sample is due to differences in genotype (Fig. 7D). The shape differences associated with PC1 indicated that TS mice (positive PC1 scores) showed wider skulls with retracted faces in comparison to WT mice (negative PC1 scores), in which the relative positions of the anatomical landmarks represented in the wireframes are associated with slender skull shapes (see Supplementary Figure S6 for anatomical reference). Thus, the shape changes represented by PC1 correspond to the main clinical features associated with Down syndrome, such as brachycephaly and midfacial retraction^54^. As shown in Fig. 7E, the skulls in TS mice (red surface) are typically shorter, wider and present substantially less developed faces as compared to WT mice (yellow surface). Significant differences due to pharmacological treatment were not detected in this analysis, as WT untreated and treated mice overlapped in the negative extreme of PC1, and TS untreated and treated mice remained in the positive extreme of PC1 (Fig. 7D). Further vice- or whiceCT analyses with higher sample sizes should be used to assess a potential effect of pharmacological modulation in TS mice.

## Discussion

To resolve the current need for a methodology to accurately phenotype the brain, ventricular system and craniofacial anatomy in small animal models, we designed several strategies to visualize the brain anatomy and the entire craniofacial complex with high resolution using a single imaging modality. As µCT has the ability to provide excellent visualization of the craniofacial bones with high resolution but needs contrast enhancement to enable the visualization of brain anatomy, we investigated three approaches involving delivery of a contrast agent, i.e. Lugol’s solution, into the mouse brain.

A first approach based on transcardial perfusion of contrast resulted in successful whole-body contrast enhancement leaving the craniofacial structures intact. However, this technique needs to be accompanied with blood–brain barrier disruption in order to achieve iodine-based contrast agent delivery into the brain parenchyma, as already pointed out by previous findings ^33^. Even then, this approach resulted in patchy enhancement of brain anatomy (Fig. 1).

We developed two other novel contrast infusion approaches that successfully achieved specific ventricular and whole-brain contrast enhancement and subsequent visualization with µCT. We named these techniques as “ventricle injection contrast-enhanced CT” (viceCT) and “whole head iodine-based contrast-enhanced computed tomography” (whiceCT).

In viceCT, the contrast agent is injected stereotactically and bilaterally into the ventricular system under terminal anesthesia. This optimized protocol resulted in successful visualization of the brain ventricular system in the context of the intact whole body and craniofacial structures using fast high-resolution ex vivo µCT (Fig. 5). A remarkable novel asset to this approach is that it enables the evaluation of ventricular volume and morphology with minimal loss of cerebrospinal fluid pressure, as the images are obtained short after euthanasia. Brain shrinkage due to sample processing was not a significant issue. ViceCT produced similar visualizations of the ventricles as compared to in vivo µMRI, although viceCT did not visualize the most ventral part of the ventricles. Bland Altman analysis revealed a disagreement of 5.39% of the mean ventricle volume derived from µMRI versus viceCT (Fig. 5, E), a difference that may not be biologically relevant. Significant differences in ventricular volume are typically at least 10%, as detected in experiments using µMRI in adult mice to study neurodegeneration, brain inflammation or Down syndrome ^43,55,56^. Using viceCT scans, it is also possible to perform detailed shape analysis of the skull, as the skeleton remains intact except for the two burr holes used for the stereotactic injection. This is thus a valid and higher resolution endpoint alternative to in vivo µMRI, the current standard modality for visualizing the intact brain ventricular system but unable to capture the cranium.

ViceCT can be subsequently complemented with whiceCT to assess brain morphology. WhiceCT is based on diceCT, the technique where different structures become enhanced on µCT through immersion of the sample in Lugol’s solution and diffusion of iodine into the tissues. WhiceCT extends diceCT capabilities by optimizing the visualization of brain parenchyma with minimal shrinkage, while maintaining the craniofacial structures intact (Fig. 3). This approach enabled for the first time the simultaneous acquisition and analysis of the detailed brain anatomy and gross craniofacial structures with fast, high-resolution µCT. After careful removal of all the soft tissues surrounding the skull, detailed analysis of craniofacial structures can also be achieved by whiceCT, at a comparable level as viceCT (Fig. 6). Regarding the mouse brain, the whiceCT protocol allowed the detailed visualization of specific internal structures, such as the cerebellum, cortex and olfactory bulbs, and has the potential to deliver ultra-detailed images of the brain using even higher resolution µCT. We illustrated here that this allows the evaluation of smaller structures or layers of interest with even greater detail, such as the Ammon’s horn, dentate gyrus or corpus callosum (Fig. 4). WhiceCT offers a better, high image quality and low-cost alternative with shorter scanning time compared to the current state-of-the-art that requires acquiring and co-registering a several hour-long ex vivo µMRI and a µCT scan to visualize skeletal structures.

A drawback of whiceCT may be that the incubation time is prolonged to 18 days, as compared to diceCT protocols for mouse brain imaging that report only 1–2 days incubation to reach homogeneous contrast enhancement ^30–32^. However, this incubation time is within an acceptable range, since more recent techniques employing PTA-enhanced µCT optimized for embryonic and early postnatal mice require even longer incubation periods in adult mice ^34^. The advantage of the whiceCT protocol that outweighs the relatively long incubation time is that the craniofacial structure is conserved. Regarding brain size comparisons, we found a smaller cerebellar volume derived from whiceCT in comparison to µMRI, which could be attributed to some degree of shrinkage specifically affecting this brain region during sample processing (Fig. 3, C). To assess the bias between both modalities, differences in resolution should also be considered, as µCT provides a much more accurate measure due to the 27.6 times smaller voxel size in our case, making whiceCT more precise.

To assess the possibility to visualize both the brain parenchyma and the ventricles simultaneously in the same scan, we observed that after performing the viceCT, the Lugol’s solution did not remain long enough in the brain ventricles to combine it with the long sample incubation required for whiceCT. However, it is possible to combine both protocols in a convenient working pipeline as a mouse can be anaesthetized to perform the viceCT protocol and after scanning, the head can be extracted to start immediately after the whiceCT protocol. The burr holes for the stereotactic injection actually enhance contrast diffusion for whiceCT. To illustrate this, we applied our vice- and whiceCT pipeline to evaluate genotype and pharmacological effects on the craniofacial and brain systems in a mouse model of Down syndrome (Fig. 7). We were able to demonstrate the capability of vice- and whiceCT to detect and quantify differences in the volume of the brain, the ventricles and the cerebellum, as well as in the skull shape applying Geometric Morphometrics on the high-resolution µCT.

### Conclusions and future directions

**Figure 8:**
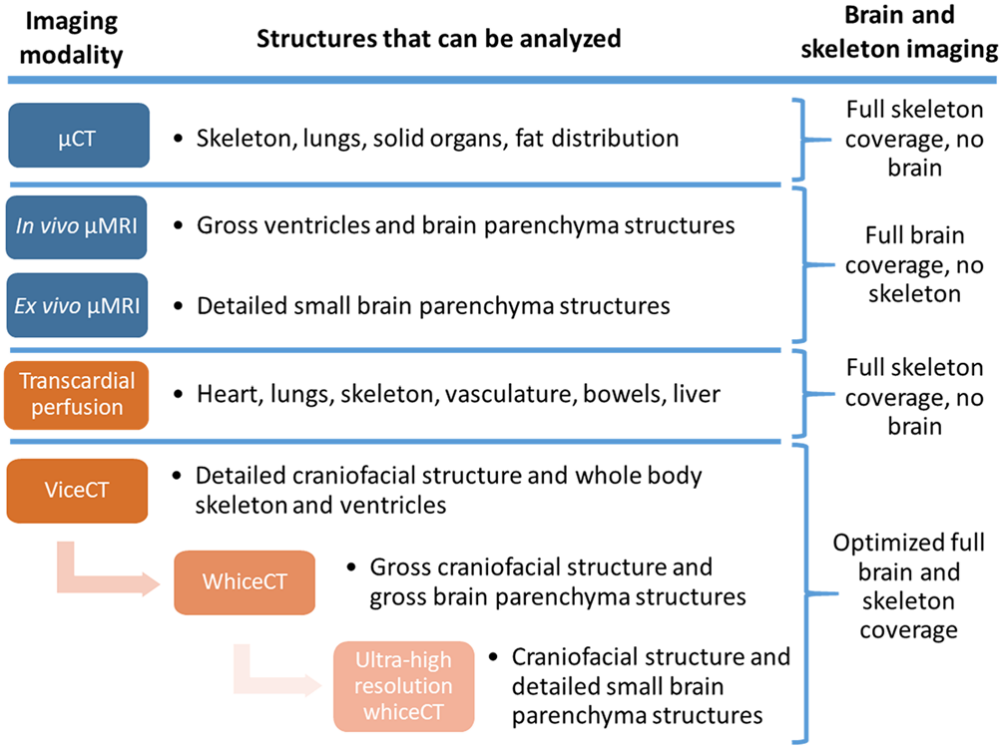
Overview of the applications of µMR and µCT-based imaging for brain and skull analysis. Left column shows the imaging modality and optional use in a sample processing and imaging pipeline. Central column shows the structures that can be analyzed using such modality independently. Right column summarizes the readouts that can be obtained using the different modalities and combinations thereof.

Typically, to visualize the brain and the skull simultaneously, it is necessary to perform a co-registration of µMRI and µCT. µMRI can deliver great contrast in the brain but is unable to visualize the skeleton, whereas µCT can visualize the skeleton together with the fat and lungs, but is unable to visualize the brain. In comparison to existing techniques, viceCT and whiceCT need only a single µCT scan to visualize the brain (whiceCT) or the ventricles (viceCT) ex vivo, maintaining the anatomical context of the brain anatomy and ventricles within the cranium (Fig. 8). Although it is still necessary to co-register the µCT scans if the brain parenchyma needs to be visualized together with the ventricles, our approach represents a step forward on simultaneous imaging of the brain and the skull (Fig. 8). An additional asset is that viceCT and whiceCT would allow destaining of the sample using sodium thiosulfate and performing histology after imaging ^25,57^. In future work, further improvements to the procedures could envisage the use of hyper-high resolution scans (≈ 1 µm) and post-hoc optimization with software for contrast enhancement such as ANKAphase ^58^. In addition, different concentrations of iodine, or different temperatures during incubation could be tested to further optimize the contrast and incubation time for samples with completely intact craniofacial anatomy.

We envisage a wide array of possible applications where this novel methodology covers an unmet need, such as when planning for targeting a specific brain region with stereotactic injection, which currently needs a brain µMRI co-registered with a skull µCT as a reference ^18,59,60^. Other examples where viceCT and/or whiceCT could be instrumental are phylogenetic and evolutionary studies ^27^, as well as studies in neuroscience research that involve precise phenotyping and experimental procedures with (transgenic) animal models of neurodevelopmental disorders with simultaneous affectation of the brain and craniofacial structure.

## Methods

### Animals and ethical statement

Wildtype (WT) mice from the KU Leuven animal facilities were used for experiments. Trisomic mice and euploid littermates were obtained from our in-house breeding colony, established from WT male and Ts65Dn females (refs. 001875 and 001924, the Jackson Laboratory Bar Harbor, ME, USA). The date of conception (E0) was determined as the day in which a vaginal plug was present. We bred two litters, one of which was administered with GTE-EGCG (Mega Green Tea Extract, Life Extension, USA) via drinking water at a concentration of 0.09 mg/mL. Epigallocatechin gallate (EGCG) crosses the placenta and reaches the embryo^61^. The treatment started prenatally at embryonic day 9 (E9) via the drinking water of the pregnant dams, and continued until the mice were scanned at 7 months of age. In total, 14 mice were generated and genotyped by PCR from ear snips. The mice were allocated in groups according to their genotype and pharmacological intervention (WT and TS, untreated and treated), which included four animals per group except in the TS untreated group, in which only one mouse survived. Mice were housed at the animal facility of KU Leuven in standard individually ventilated cages (40 cm long × 25 cm wide × 20 cm high) under a 12 h light/dark schedule in controlled environmental conditions of humidity (50–70%) and temperature (22 ± 2 °C) with food and water supplied ad libitum. Water intake was monitored by cage. All procedures complied with all local, national and European regulations and were authorized by the Animal Ethics Committee of KU Leuven (ECD approval number P004/2016).

### Contrast agent

Lugol’s solution was purchased from Sigma Aldrich (St. Louis, USA), with a concentration of (KI) 0.67–0.71% and (I) 0.33–0.37%.

### Transcardial perfusion

Adapting the protocol described in ^62^, mice were terminally anesthetized using 3 µL/g of Dolethal (0.2 mg/µL pentobarbital, Vétoquinol) until no twitch response was obtained from a toe pinch. After sedation, each mouse was secured in supine position by gently taping the forepaws and hind paws to a pintable inside a chemical fume hood. Then, an incision was made through the skin with surgical scissors along the thoracic midline from just beneath the xiphoid process up to the clavicle. Two additional skin incisions were made from the xiphoid process along the base of the ventral ribcage laterally. Next, the two skin flaps were reflected rostrally and laterally to completely expose the thoracic field, and the xiphoid process was grasped with blunt forceps and raised slightly to insert pointed scissors. The thoracic musculature and ribcage were then cut between the breastbone and medial rib insertion points and the incision was extended rostrally to the level of the clavicles. Afterwards, the diaphragm was separated from the chest wall on both sides with scissor cuts, and the reflected ribcage was pinned with 18G needles to expose the heart and the rest of thoracic organs. Then, a 24G needle was inserted in the left ventricle. Immediately after, the right atrium was cut with scissors and infusion began at the first sign of blood flow.

Mice were perfused using saline, paraformaldehyde (PFA, 4% in PBS) and Lugol’s solution. To remove the blood from the mouse, we first perfused saline until the fluid exiting the right atrium was entirely clear (step 1); then, we perfused 20 to 30 mL of PFA to fix the tissue (step 2); and finally, to increase the contrast of the soft tissues, we perfused Lugol’s solution until the liver and paws were colored (step 3). The perfusion steps were optimized and altered according to Supplementary Table S1.

### WhiceCT sample preparation

Mouse heads were extracted by decapitation several vertebrae below the base of the skull. Then, two burr holes were drilled in the skull to facilitate diffusion (a step not necessary if performed after viceCT, as two drill holes are already present from the stereotactic injection). Next, the heads were fixed in 4% PFA at 4 °C and the soft tissue of the head was removed. Then, the samples were incubated in Lugol’s solution in 50 mL centrifuge tubes at 4 °C throughout sample processing.

### Stereotactic injection for viceCT

Mice were terminally anesthetized with an intra-peritoneal injection of 3 µL/g of Dolethal and were placed in a stereotactic head frame (Stoelting, Wood Dale, IL, USA) for stereotactic injections as described before ^59^, with minor adaptations. The skull was exposed via a midline incision in the skin. The skull flat position was achieved by placing bregma and lambda in the same horizontal plane by adjusting the vertical tooth bar. One or two burr holes were made at the planned entry location depending on the experimental approach. Next, a 30 G needle loaded with contrast fluid (U-100, Terumo, Leuven, Belgium) was mounted on the stereotactic arm, positioned at bregma and then translated antero-posteriorly and medio-laterally along to the planned trajectory to target the right (coordinates (mm): antero-posterior (AP): − 0.2; medio-lateral (ML): − 1.0; dorso-ventral (DV): − 1.8)) and left (coordinates (mm): antero-posterior (AP): − 0.2; medio-lateral (ML): + 1.0; dorso-ventral (DV): − 1.8)) lateral ventricles. Finally, after perforation of the dura mater, the needle was lowered until reaching the planned coordinates in a single motion in approximately ten seconds. After injection, the needle was kept in place for a minute to allow liquid deposition, and then gently removed from the brain.

For the unilateral approach, a stereotactic injection of 100 or 50 µL was performed in the right lateral ventricle with an injection rate of 0.33 µL/s and an injection time of 2 min 30 s. For the bilateral injection approach, 40 µL was injected in each of the lateral ventricles with an injection rate of 0.22 µL/s and an injection time of 3 min.

### Micro-computed tomography (µCT)

Whole-body mice or heads (in batches of 3) were scanned with the SkyScan 1278 (Bruker Micro-CT, Kontich, Belgium) using the optimized parameters specified in Supplementary Table S2. For the ultra-high-resolution scan, the heads were individually maintained in a humid environment by placing two sources of water next to the sample and covering it with parafilm to avoid the sample from drying and consequent shrinking, and scanned using the SkyScan 1076 (Bruker Micro-CT, Kontich, Belgium).

### Micro-magnetic resonance (µMR)

In vivo µMRI measurements were performed using a 9.4 T Bruker Biospec small animal µMR scanner (Bruker Biospin, Ettlingen, Germany; 20 cm horizontal bore) equipped with actively shielded gradients (600 mT m^−1^). A quadrature radio-frequency resonator (inner diameter 7.2 cm, Bruker Biospin) was used for transmission of radiofrequency pulses and decoupled to a brain surface coil (quadrature shaped surface coil optimized for mouse brain scanning, Bruker Biospin). In vivo anatomical images were obtained through a 3D T2 weighted RARE sequence (TR/TE: 1,500/ 43.44 ms; RARE factor: 16; FOV: 20 × 20 × 20 mm^3^; matrix 128 × 128 × 128; acquisition time 20 min).

Ex vivo µMRI images were acquired four days after performing a transcardial perfusion with a 10 mM Gd-DTPA solution, following a previously published protocol ^63^. The images were acquired using a 3D FLASH pulse sequence: 11 ms TE, 75 ms TR, 1.6 × 3.2 × 1.6 cm FOV, 256 × 512 × 256 matrix yielding 62.5 μm isotropic resolution, 4 averages, 1 repetition resulting in a total acquisition time of 12 h 17 min.

### Density calibration and image quality assessment

To convert grey values into Hounsfield units (HU), the mean gray value within a volume of interest (VOI) of ten slices placed in the ventricles or the parenchyma (CTAn software, Bruker Micro-CT, Kontich, Belgium) was converted from a calibration line obtained from scanning a water-in-air phantom using the same settings, in which the mean gray value of air was set to − 1,000 HU and the mean gray value of water to 0 HU.

The signal to noise ratio (SNR) for viceCT was computed in CTAn by calculating the ratio between the mean gray value within a VOI of ten slices placed in the ventricles or the parenchyma and the standard deviation of the same VOI. For the µMR images, SNR was calculated as the mean gray value of the ventricle or the parenchyma divided by the standard deviation of the background noise. The mean gray value of the brain ventricles and the brain parenchyma were computed by the weighted average of the mean gray value within a region of interest (ROI) placed in those regions in five slices. The standard deviation of the background noise was computed as the weighted average of the standard deviation within an ROI placed in the background in five slices multiplied by 1.53, to adjust for the differences in the Rayleigh noise distribution.

The contrast to noise ratio (CNR) was computed in CTAn from viceCT data by calculating the difference in mean gray value within a VOI of ten slices placed in the ventricles and the mean gray value within a VOI of ten slices placed in the parenchyma and dividing it by the standard deviation of the VOI placed in the parenchyma. For the µMR images, CNR was calculated as the difference between the mean gray value of the ventricle and the parenchyma, divided by the standard deviation of the background noise, computed as explained above.

### Data processing

To segment the brain, ventricles and cerebellum, the µMRI data were first converted to Analyze file format using ImageJ (1.52d, National Institute of Health), then loaded into Amira (v5.2.1, Visualization Sciences Group, FEI) together with the µCT data. Then, each region was manually segmented by drawing a region of interest covering those structures on each image slice and the volume reported.

### Statistical analysis

We performed all the statistical tests using GraphPad Prism (v5.04, GraphPad Software, San Diego, California USA).

The Bland Altman test is a method to quantify the agreement between two quantitative measurements ^64^. In this method, the bias between two measured volumes is established as the average of the difference between the volumes measured by the two methods. The limits of agreement are estimated as the 95% limits of agreement based on the standard deviation of the bias. The mean volume of the analyzed structure was calculated as the mean of all the measurements taken with both µMRI and µCT. The percentage shift of the bias was estimated as the ratio between the bias over the mean volume of the analyzed structure, as $\frac{{Bias \left( {average \,difference \,between\, two \,techniques} \right)}}{Mean \,volume\, of \,the\, analyzed \,structure} \times 100$. The percentage shift associated with the upper and lower 95% limits of agreement was estimated using the same formula but using in the numerator the scores of the upper and lower limits calculated from the standard deviation of the bias. These values indicated the range of variation of the percentage shift in individual measurements between the two methods.

Uncorrected Dunn’s test was used to assess if the difference in the volume of the whole brain, cerebellum and ventricles were significant.

### Shape analysis

We quantitatively compared the skull morphology of WT mice with and without GTE-EGCG and TS treated mice using a quantitative shape analysis ^65^. The analysis was based on the 3D coordinates of 27 anatomical skull landmarks registered using Amira on the µCT isosurfaces obtained from the viceCT protocol (Supplementary Figure S6). To assess shape variation we performed a Generalized Procrustes Analysis (GPA) followed by a Principal Component Analysis (PCA) using MorphoJ ^66^. Skull shape variation was assessed by creating a morphospace based of the two first PCs, which explain the largest percentages of morphological variation within the sample.

## Supplementary information

Supplementary Information 1.

**Video 1. Visualization of the skull and brain in 3D from a sample scanned with the optimal whiceCT protocol.** The video shows that the skull is visible in 3D from a whiceCT scan and that different regions of the brain are discernable within the skull.

**Video 2. Visualization of the skull and the brain ventricles in 3D from a sample scanned with the optimized viceCT protocol.** The video shows that the entire body and skeleton of the mouse are visible in 3D from a viceCT scan and that the ventricles are discernable within the skull.

## Data Availability

Because of data size, data is available upon simple reasonable request to the corresponding author.
