# Supplementary material for: ViceCT and whiceCT for simultaneous high-resolution visualization of craniofacial, brain and ventricular anatomy from micro-computed tomography
Source: Sci Rep. 2020 Oct 30;10:18772. doi: 10.1038/s41598-020-75720-3 (PMC7599226; doi:10.1038/s41598-020-75720-3)
Supplement: Supplementary file 1 — Supplementary Information 1. [file 41598_2020_75720_MOESM1_ESM.pdf]

SUPPLEMENTARY MATERIAL

ViceCT and whiceCT for simultaneous high-resolution visualization of craniofacial, brain and ventricular anatomy from micro-computed tomography

Authors & Affiliations

Sergi Llambrich[1,2], Jens Wouters[1,2], Uwe Himmelreich[1,2], Mara Dierssen[3,4], James Sharpe[5], Willy Gsell[1,2], Neus Martínez-Abadías[6], Greetje Vande Velde*[1,2]

[1]Biomedical Imaging, Department of Imaging and Pathology, Faculty of Medicine, KU Leuven, Leuven, Belgium

[2]Molecular small animal imaging Centre (MoSAIC), KU Leuven, Leuven, Belgium

[3]Centre for Genomic Regulation (CRG) The Barcelona Institute of Science and Technology, 08003 Barcelona, Spain

[4]Universitat Pompeu Fabra (UPF), 08003 Barcelona, Spain

[5]EMBL Barcelona, European Molecular Biology Laboratory, Barcelona, Spain Institució Catalana de Recerca i Estudis Avançats (ICREA), Barcelona, Spain

[6]GREAB-Research Group in Biological Anthropology. Department of Evolutionary Biology, Ecology and Environmental Sciences, BEECA. Universitat de Barcelona, Barcelona, Spain

Corresponding author:

Greetje Vande Velde
Greetje.vandevelde@kuleuven.be
Herestraat 49 O&N1 box 505
B-3000 Leuven, Belgium
+3216330924

33

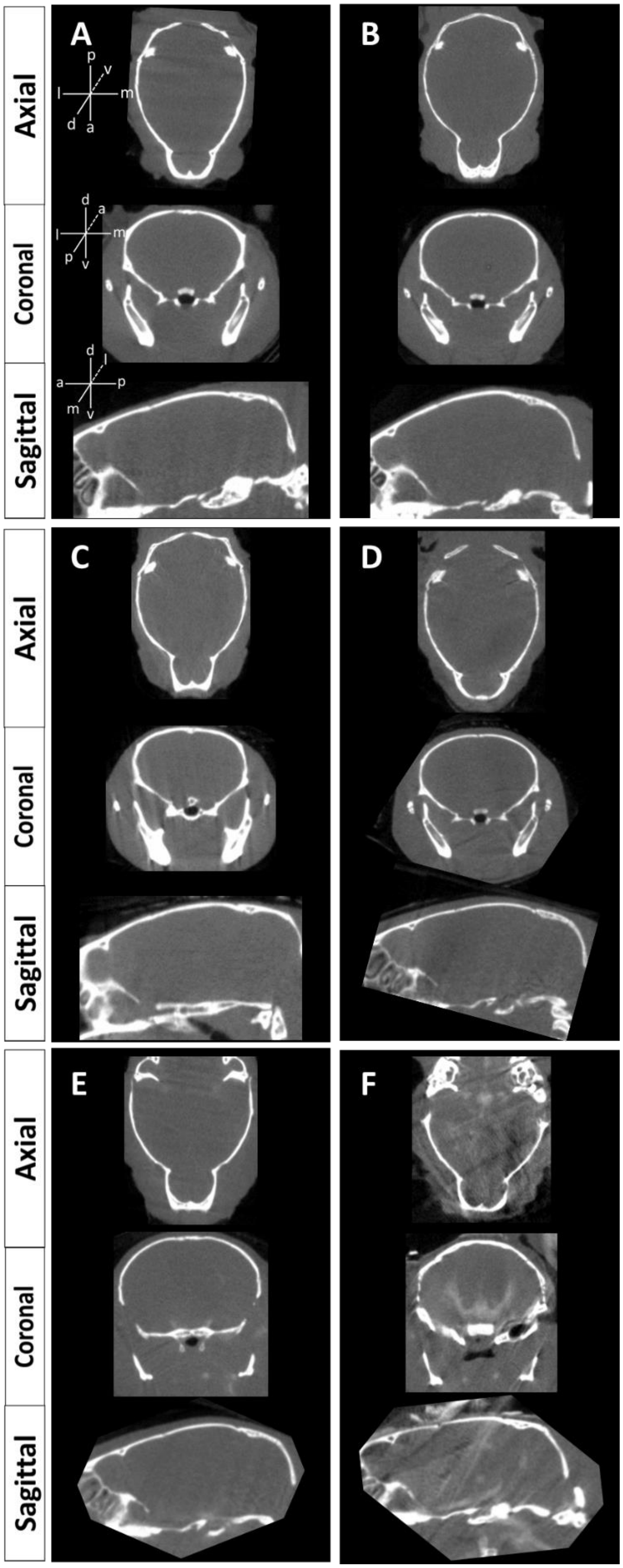

Supplementary Figure S1. µCT of mouse heads after transcardial perfusion with Lugol's solution. Each panel corresponds to the reconstructed image obtained using the protocol with the same letter in Supplementary Table S1. In each scan, the craniofacial structures are visible, but in panels A-E there is no brain contrast at all. Protocol F (Panel F) resulted in non-homogeneous contrast in the brain (note: shadowing artifacts are due to a metallic ear tag).

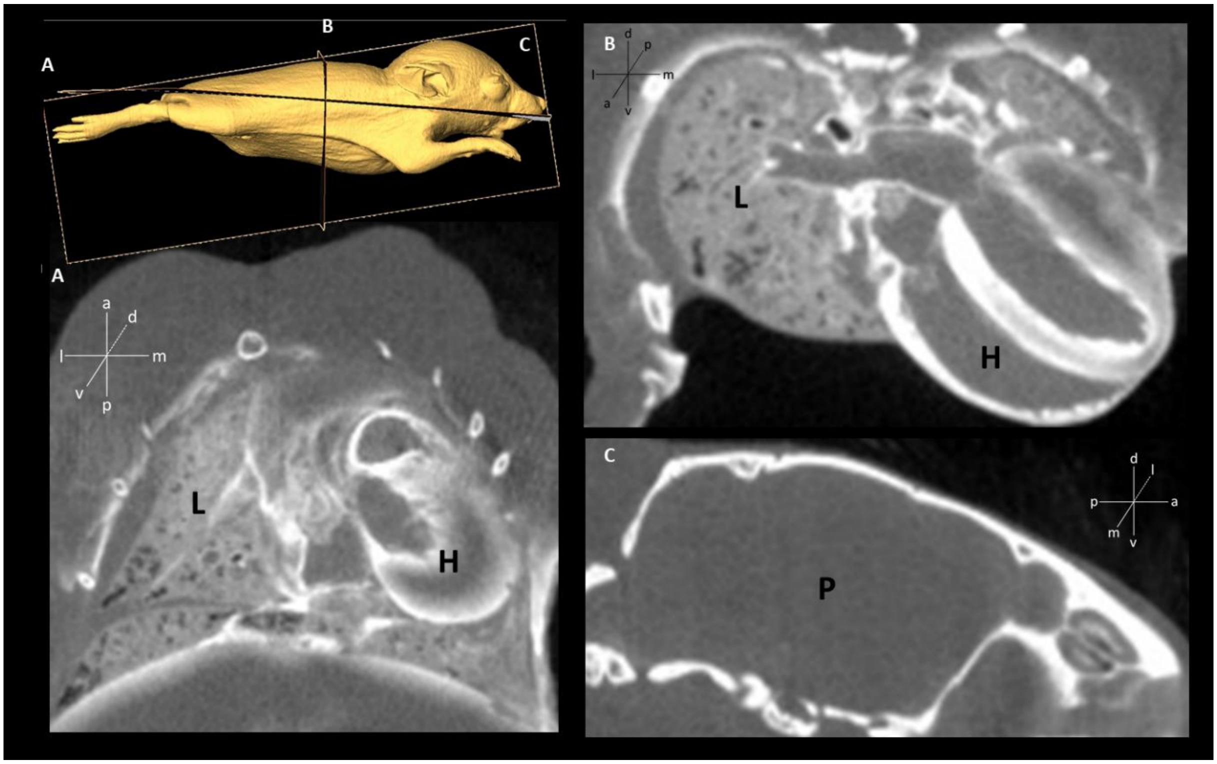

Supplementary Figure S2. Whole-body µCT upon transcardial perfusion of Lugol's solution results in contrast enhancement in body organs but not in brain. Top left corner shows the surface rendering of a reconstructed whole body µCT of a mouse perfused according to Supplementary Table S1 protocol G, indicating the location of planes shown in panels A, B and C. **(A)** Axial and **(B)** coronal view of the thorax. µCT scans revealed that transcardial contrast perfusion resulted in immediately detectable contrast enhancement in soft tissue structures such as the lungs (L) and heart (H), making it possible to visualize the cardiac muscles, pericardial structures and the lung architecture with great detail. **(C)** Sagittal view of the head. The skull is clearly visualized, but no contrast can be detected in the brain parenchyma (P).

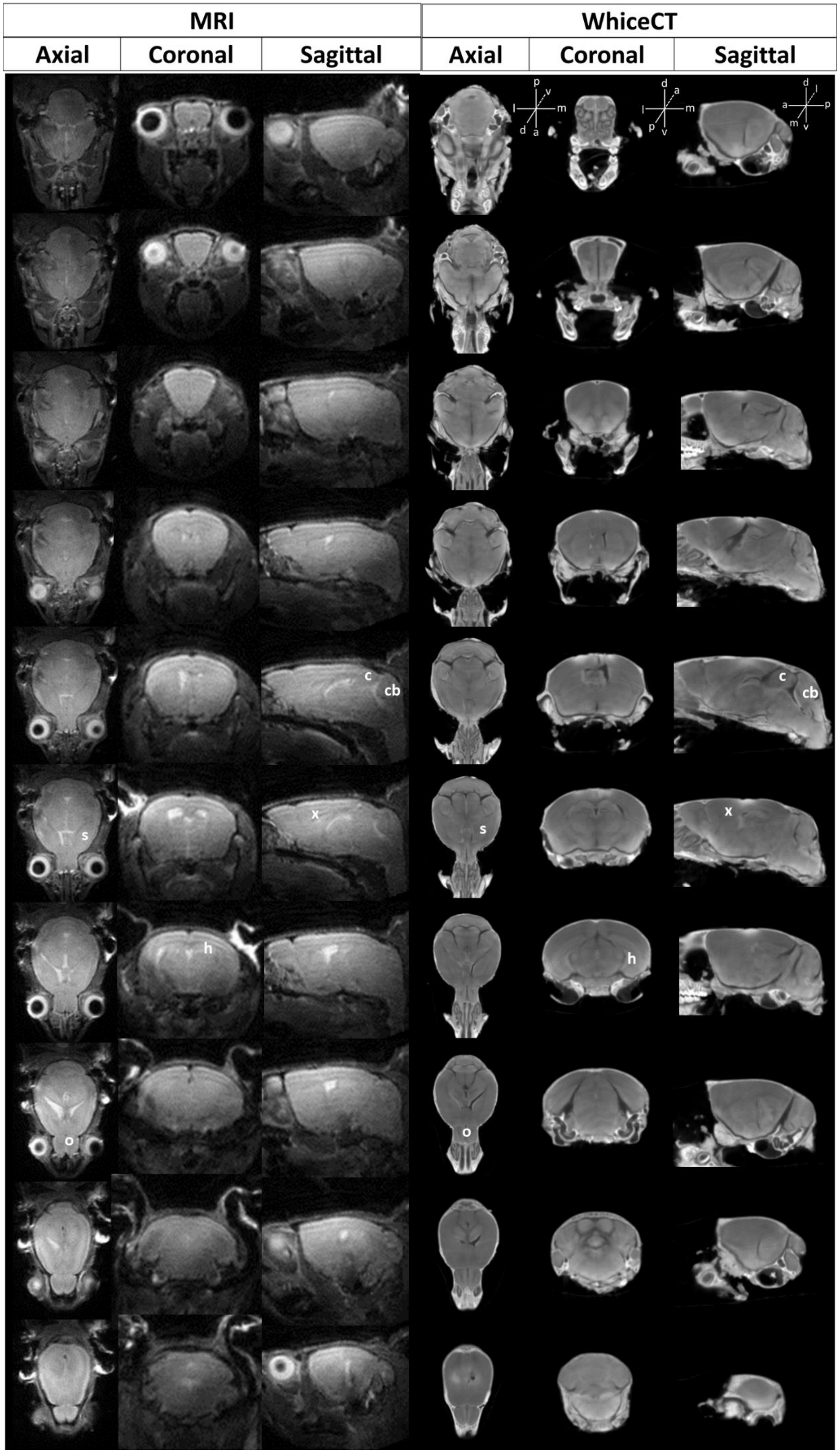
MRI
WhiceCT
Axial
Coronal
Sagittal
Axial
Coronal
Sagittal
c
cb
s
x
h
o

Supplementary Figure S3. Comparison of µMRI and whiceCT from multiple planes. The striatum (s), hippocampus (h), isocortex (x), olfactory bulbs (o), cerebellum (cb) and colliculi (c) are indicated.

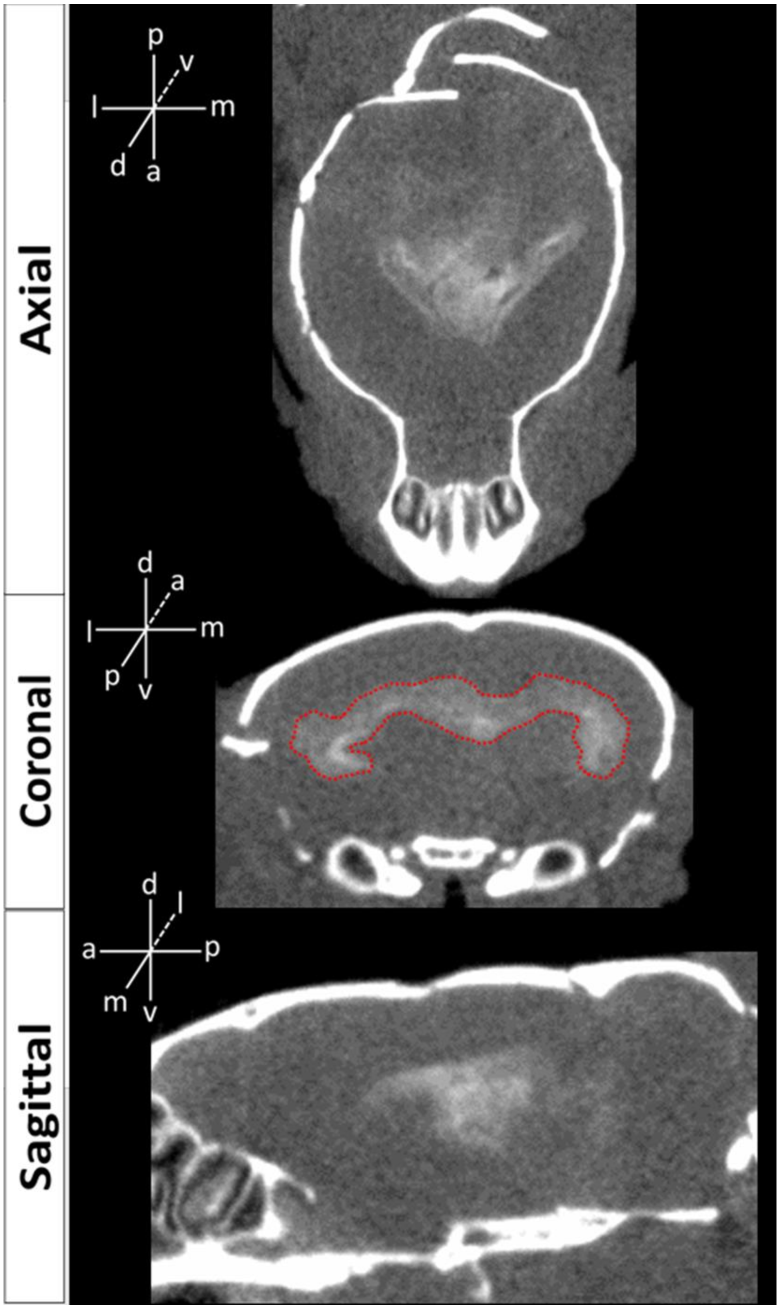

Supplementary Figure S4. µCT shows overflow of the contrast agent from the ventricles after unilateral injection of 100 µL of Lugol's solution and possible alterations of the ventricular anatomy. Unilateral stereotactic injection of 100 µL of Lugol's solution into the right lateral ventricle resulted in the contrast not being confined in the ventricles (white arrows). The red dotted line in the coronal plane outlining the

ventricles indicates that the high pressure during contrast administration could have altered the ventricular anatomy. Axial (top), coronal (middle) and sagittal (bottom) planes.

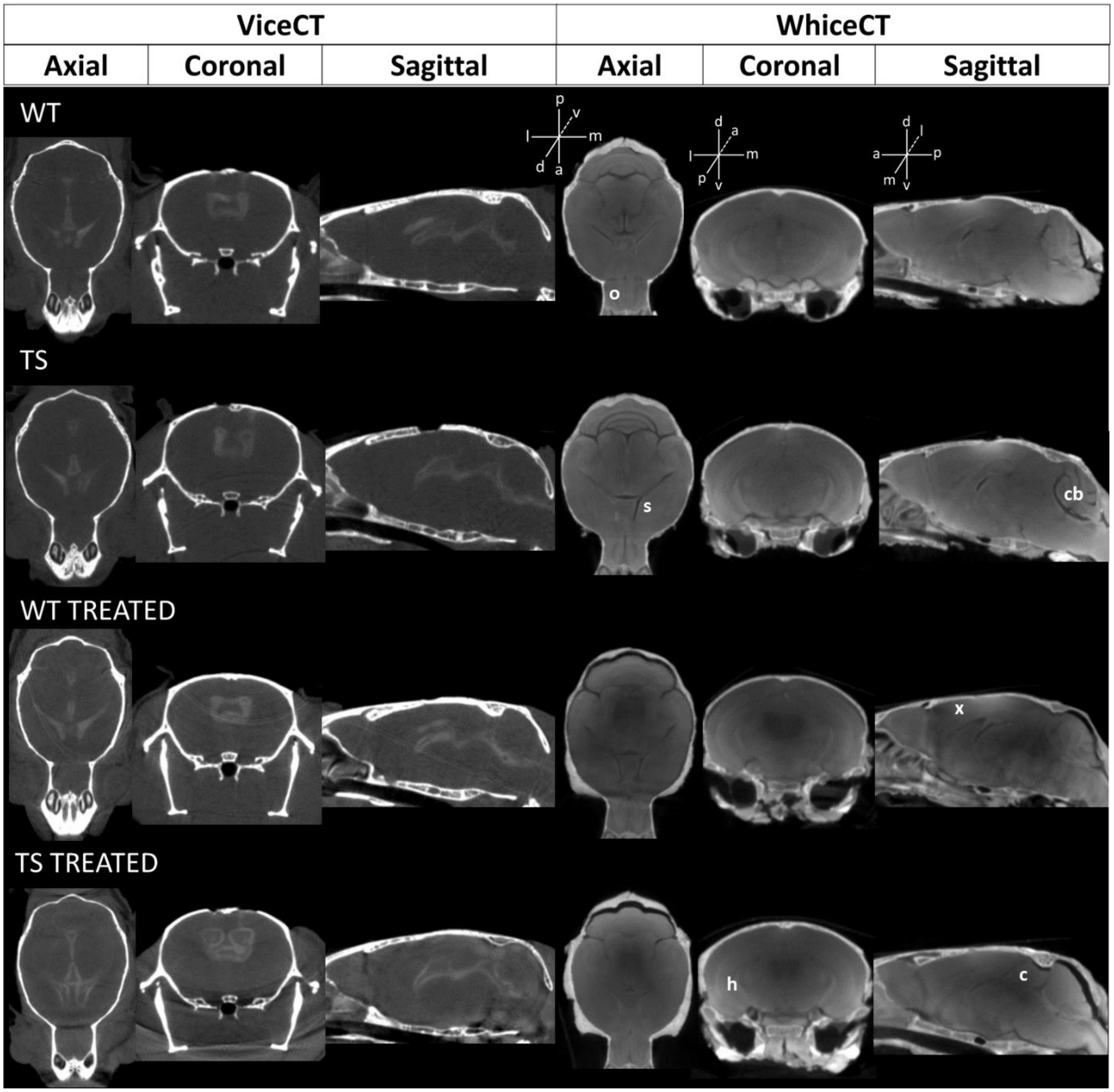

Supplementary Figure S5. Comparison between wildtype and trisomic mice, treated or not with GTE-EGCG, using viceCT and whiceCT. Wildtype mice untreated (WT), trisomic mouse treated (TS), wildtype mice treated with GTE-EGCG as a modulatory agent (WT TREATED) and trisomic mice treated with GTE-EGCG as a modulatory agent (TS TREATED) are shown. The striatum (s), hippocampus (h), isocortex (x), olfactory bulbs (o), cerebellum (cb) and colliculi (c) are indicated in the whiceCT images.

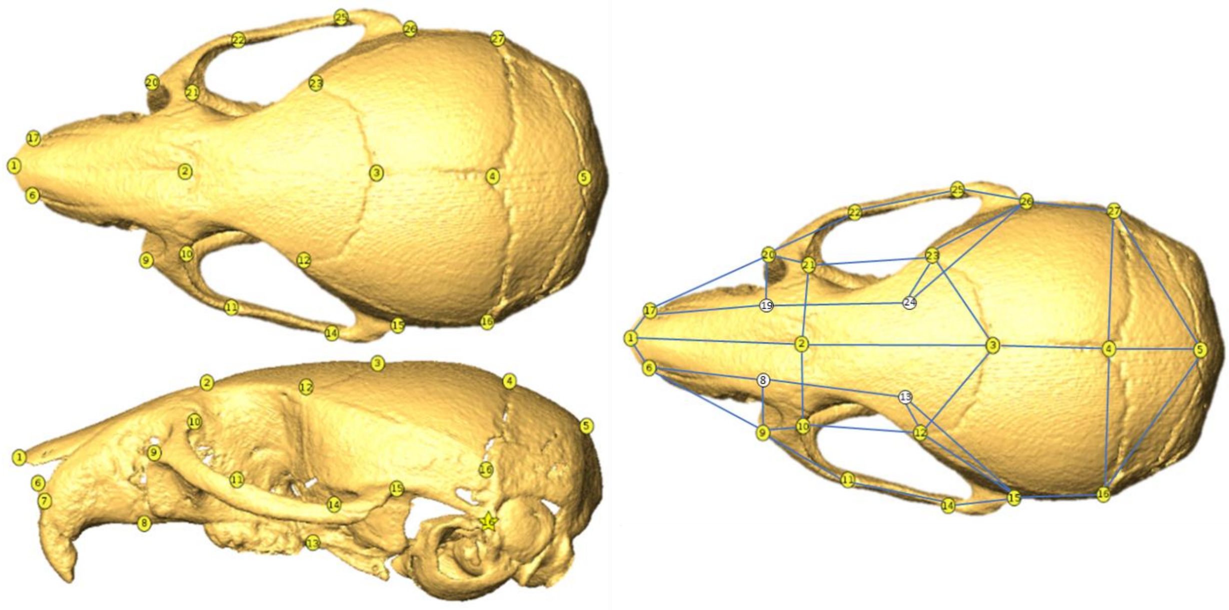

Supplementary Figure S6. Set of 27 anatomical landmarks used to characterize adult mouse craniofacial morphology. Left: µCT 3D surface renderings of a mouse skull with the position of each landmark. Right: overlay of skull reconstruction with wireframe used in Fig. 7D**.** See Supplementary Table S3 for precise anatomical definitions.

Protocol	Step 1	Step 2	Step 3	Step 4	Brain contrast
A	Saline	PFA 4%	Lugol	-	No
B	Saline	50/50 Lugol and PFA 4%	-	-	No
C	Saline	Lugol 20 min	PFA 4%	-	No
D	Saline	Lugol 20 min	PFA 4% 48h diffusion	-	No
E	Saline	Lugol 20 min	Saline	PFA 4% 48h diffusion	No
F	-	Lugol 20 min	PFA 4% 48h diffusion	-	Yes, patchy
G	-	Lugol 50 min	PFA 4% 48h diffusion	-	No
H	Mannitol Intra Peritoneal Injection	30 min wait	Lugol 30 min	-	Yes, patchy

Supplementary Table S1. Overview of the different protocols tested for transcardial perfusion of the contrast.

Method	Source energy (Kv)	Filter	Current (µA)	Exposure time (ms)	Averages	Step increment (˚)	Total angle (˚)	Time	Voxel size (µm³)	System
Transcardial perfusion	50	1 mm Al	918	55	3	0.6	360	6 min	51.62	SkyScan 1278
WhiceCT	50	0.5 mm Al	996	70	6	0.6	360	11 min	51.62	SkyScan 1278
Ultra-high resolution whiceCT	50	0.5 mm Al	179	2200	6	0.6	360	3 h 3 min	9.05	SkyScan 1076
ViceCT	59	1 mm Al	831	55	6	0.6	360	11 min	51.62	SkyScan 1278

Supplementary Table S2. Overview of optimized µCT scanning parameters used for the different contrast administration protocols.

Landmark number	Anatomical definition
1	Tip of the nasal bone
2	Intersection of nasal and frontal bones
3	Intersection of frontal and parietal bones
4	Intersection of parietal and interparietal bones
5	Intersection of interparietal and occipital bones
6	Anterior-most point on intersection of premaxillae and nasal bones (left)
7	Center of alveolar ridge over maxillary incisor (left)
8	Most inferior point on premaxilla-maxilla suture (left)
9	Anterior notch on frontal process lateral to infraorbital fissure (left)
10	Intersection of frontal process of maxilla with frontal and lacrimal bones (left)
11	Intersection of zygomatic process of maxilla with zygoma (left)
12	Frontal-squamosal intersection at temporal crest (left)
13	Intersection of maxilla and sphenoid on inferior alveolar (left)
14	Intersection of zygoma with zygomatic process of temporal (left)
15	Intersection of squamosal body to zygomatic process of squamosal (left)
16	Intersection of parietal, temporal and occipital bones (left)
17	Anterior-most point on intersection of premaxillae and nasal bones (right)
18	Center of alveolar ridge over maxillary incisor (right)
19	Most inferior point on premaxilla-maxilla suture (right)
20	Anterior notch on frontal process lateral to infraorbital fissure (right)
21	Intersection of frontal process of maxilla with frontal and lacrimal bones (right)

22	Intersection of zygomatic process of maxilla with zygoma (right)
23	Frontal-squamosal intersection at temporal crest (right)
24	Intersection of maxilla and sphenoid on inferior alveolar (right)
25	Intersection of zygoma with zygomatic process of temporal (right)
26	Intersection of squamosal body to zygomatic process of squamosal (right)
27	Intersection of parietal, temporal and occipital bones (right)

Supplementary Table S3. Definitions of landmarks used in adult mice to characterize craniofacial morphology. Landmarks were collected on µCT 3D reconstructions of the craniofacial skeleton of adult mice of the Ts65Dn Down syndrome mouse model. See Supplementary Figure S6 for anatomical reference.

ViceCT	Mean grey value			Standard Deviation			SNR		CNR	HU		
Mouse	Ventricle	Parenchyma	Background	Ventricle	Parenchyma	Background	Ventricle	Parenchyma	Ventricle	Ventricle	Parenchyma	Shift
1	103.05	42.24	0	7.18	2.91	0	14.35	14.51	20.89	1934	203	-1731
2	79.58	42.53	0	4.97	2.15	0	16.03	19.75	17.20	1266	211	-1055
3	72.21	40.12	0	3.16	1.49	0	22.83	26.95	21.56	1056	142	-914
4	83.62	41.51	0	3.56	1.72	0	23.52	24.17	24.52	1381	182	-1199
5	71.13	41.43	0	2.40	1.15	0	29.64	36.10	25.88	1025	180	-846
6	77.42	40.91	0	3.61	1.48	0	21.42	27.72	24.75	1204	165	-1040
						Average	21.30	24.87	22.47	1311	180.33	-1131
						Std Dev	5.05	6.74	2.95	304	22.81	291

ViceCT	Mean grey value	HU
Timepoints	Ventricle	Ventricle
1	99.87	1573
2	96.52	1487
3	71.00	861

µMRI	Mean grey value			Standard Deviation			SNR		CNR
Mouse	Ventricle	Parenchyma	Background	Ventricle	Parenchyma	Background	Ventricle	Parenchyma	Ventricle
1	7937.00	5560.47	236.15	351.02	282.69	193.46	41.03	28.74	12.28
2	8572.30	7160.93	822.58	822.09	463.60	348.53	24.60	20.55	4.05
3	9215.47	5578.07	727.76	639.93	263.52	241.62	38.14	23.09	15.05
4	6783.38	4861.69	298.47	324.15	179.50	170.27	39.84	28.55	11.29
5	8358.65	6235.10	413.10	340.27	220.56	125.05	66.84	49.86	16.98
6	10723.25	6212.19	641.65	408.16	309.95	293.53	36.53	21.16	15.37
						Average	41.16	28.66	12.50
						Std Dev	12.69	10.02	4.24

Supplementary Table S4. Image quality assessment. Values of the mean gray value, standard deviation, signal to noise ratio (SNR), contrast to noise ratio (CNR) and Hounsfield Units (HU). Top table shows the values for the viceCT scans performed to cross-validate with µMRI (bottom table). Middle table shows the values for the longitudinal viceCT scan to assess contrast stability.
